# Tailoring the Crystallization Behavior of Mixed Lead‐Tin Mixed‐Halide Perovskites for Optimal‐Bandgap Solar Cells

**DOI:** 10.1002/advs.202520948

**Published:** 2025-11-19

**Authors:** Lana M. Kessels, Willemijn H. M. Remmerswaal, Nick R. M. Schipper, Laura Bellini, Henry Kwan, Martijn M. Wienk, René A. J. Janssen

**Affiliations:** ^1^ Molecular Materials and Nanosystems and Institute for Complex Molecular Systems Eindhoven University of Technology P.O. Box 513 Eindhoven 5600 MB The Netherlands; ^2^ Dutch Institute for Fundamental Energy Research De Zaale 20 Eindhoven 5612 AJ The Netherlands

**Keywords:** film morphology, metal‐halide perovskites, optimal bandgap, passivation, quasi‐Fermi level splitting

## Abstract

Incorporating bromide into metal‐iodide perovskites is a commonly used approach for widening the bandgap of lead‐halide perovskites. Here, mixing of iodide and bromide is explored in narrow‐bandgap lead‐tin perovskites to create a Cs_0.1_FA_0.6_MA_0.3_Pb_0.5_Sn_0.5_I_2.5_Br_0.5_ perovskite composition, achieving the optimal bandgap of 1.34 eV for single‐junction solar cells. Introducing bromide into the precursor solution, markedly influenced film formation and resulted in singular 40 µm‐sized perovskite crystals. Supported by in situ absorption measurements, it is found that the delay time between starting the spin‐coating of the perovskite precursor and depositing the antisolvent is key in controlling the film morphology. By drastically reducing this delay time, homogenous nucleation is induced and smooth closed films are obtained. The Cs_0.1_FA_0.6_MA_0.3_Pb_0.5_Sn_0.5_I_2.5_Br_0.5_ perovskite do not show signs of light‐induced halide segregation during prolonged illumination. Using ammonium thiocyanate (NH_4_SCN) as additive in the precursor solution, the grain size could be further controlled. In solar cells, NH_4_SCN improved reproducibility and decreased hysteresis is observed. Applying passivation to reduce non‐radiative recombination at the perovskite ‐ electron transport layer interface and optimizing the device configuration results in a power conversion efficiency of 19.0%. This is among the highest for perovskites in the 1.3−1.4 eV bandgap range reported to date.

## Graphical abstract

Carefully tuning the composition of mixed lead‐tin mixed iodide‐bromide perovskites results in an optimal‐bandgap semiconductor for solar energy conversion. Homogenous crystallization of this perovskite involves a short time delay between deposition of the precursor solution and applying an antisolvent. Using a bulk additive and top surface passivation to increase grain size and reduce non‐radiative recombination, the power conversion efficiency attains 19%.

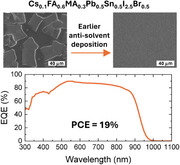

## 1 Introduction

Metal halide perovskites are suitable for high efficiency solar cells with power conversion efficiencies (PCEs) over 26%.^[^ ^1^, ^2^ ^]^ The majority of the high‐performing perovskite devices use semiconductors with a bandgap of ≈1.5 eV and are often based on compositions consisting of lead as divalent metal ion, iodide as halide, and a mixture of formamidinium (FA) and cesium as monovalent ions (Cs*_x_* FA_1−_ *_x_* PbI_3_). Further performance improvements can be achieved by reducing the bandgap to 1.34 eV, which corresponds to the optimal bandgap for single‐junction solar cells obtained from the detailed‐balance limit. However, research and, hence, the performance of perovskite solar cells with bandgaps in the 1.3−1.4 eV region is lagging behind.^[^ ^3^ ^]^ One possibility to reach the optimal bandgap is substituting lead by tin, which reduces the bandgap to ≈1.4 eV. Unfortunately, the rapid degradation due to oxidation of tin and poor film morphology currently still limit the performance to 15%.^[^ ^4^, ^5^ ^]^ By alloying tin with lead, narrower bandgaps (down to 1.25 eV) have been achieved, while also being slightly more robust to oxidation. Tin fluoride (SnF_2_) and several other additives are used to further regulate crystallization and mitigate oxidation, therewith achieving devices with PCEs up to 23%.^[^ ^6^, ^7^, ^8^ ^]^ For the ideal‐bandgap range, Liang et al. have used a FA_0.8_MA_0.2_Pb_0.8_Sn_0.2_I_3_ (MA is methylammonium) perovskite to reach a bandgap of 1.33 eV and a high PCE of 22.5%.^[^ ^9^ ^]^ However, when the lead‐tin ratio deviates too far from 50/50, it has also been reported that morphological issues appear.^[^ ^10^, ^11^ ^]^

It is well‐established that mixed‐halide compositions, i.e., bromide and iodide, in lead‐based perovskites widen the bandgap. Over the last years, PCEs of solar cells in the 1.5–1.9 eV bandgap range have rapidly increased.^[^ ^3^ ^]^ Only a few research groups reported on mixed‐halide compositions for mixed‐metal perovskites. Lee & Kang showed that incorporating small amounts of bromide (*x* = 0.2−0.4) in a MAPb_0.4_Sn_0.6_I_3‐_ *_x_* Br*_x_* perovskite can improve the crystallinity of these Sn‐rich perovskites, while minimizing a shift in bandgap. When the bromide concentration was further increased (*x* = 0.5−0.8), the bandgap widened. For *x* = 0.4 (1.26 eV), the highest PCE of 12.1% was achieved.^[^ ^12^ ^]^ While mainly focusing on wide bandgaps for tandem applications, Yang et al. investigated a series of MAPb_1‐_ *_x_* Sn*_x_* (I_0.6_Br_0.4_)_3_ perovskites. They noticed that the incorporation of tin reduced the halide segregation that is often seen in full‐lead bromide‐rich films. By increasing the tin content to *x* = 0.50–0.75, they reached bandgaps in the range of 1.2−1.4 eV. However, the layers became inhomogeneous and high concentrations of pinholes were detected, leading to poorly performing devices.^[^ ^13^ ^]^ Lee et al. introduced bromide into an inorganic CsPb_0.4_Sn_0.6_I_3‐_ *_x_* Br*_x_* perovskite and noticed that *x* = 0.6 (1.35 eV) lead to a higher crystallinity and denser morphology, thereby suppressing trap‐assisted recombination.^[^ ^14^ ^]^ The more stable inorganic structure achieved PCEs of 12.4% with high open‐circuit voltage (*V*_OC_) and fill factor (FF) of 0.86 V and 0.75, respectively. However, the short‐circuit current density (*J*_SC_) was underperforming at 19.2 mA cm^−2^. Especially the near‐infrared region was not sufficiently absorbed, stemming from morphological issues.^[^ ^14^ ^]^

It has become clear that the fabrication of mixed‐metal mixed‐halide perovskites leads to complex crystallization pathways, and controlling these will advance the performance of optimal‐bandgap perovskites. Currently, a wide range of spin‐coating procedures and antisolvent deposition times are used, ranging from early application within 5 s to longer waiting times of 45 s, where some even do not use any anti‐solvent at all (Table S1, Supporting Information). Here, we show how different antisolvent timing drastically changes the film morphology. By modifying a spin‐coating procedure that is successful for preparing mixed‐Pb‐Sn full‐iodide perovskites, the fast spontaneous inhomogeneous crystallization of a Cs_0.1_FA_0.6_MA_0.3_Pb_0.5_Sn_0.5_I_2.5_Br_0.5_ perovskite can be circumvented. Significantly reducing the time between deposition of the perovskite precursor solution and application of the antisolvent to induce crystallization allows a laterally uniform nucleation of the perovskite film to be achieved, resulting in a closed and homogeneous perovskite absorber layer with the optimal bandgap of 1.34 eV. Ammonium thiocyanate (NH_4_SCN) is used as a bulk additive leading to increased crystallite sizes, less hysteresis, and more stable devices under operating conditions. Devices were optimized using 1,2‐ethylenediammonium diiodide (EDAI_2_) for interface passivation and a thin hole‐transporting layer to reduce parasitic absorption, yielding a PCE of 19.0%, which is the highest reported efficiency for mixed‐metal, mixed‐halide perovskites in the 1.3 to 1.4 eV region. Interestingly, Cs_0.1_FA_0.6_MA_0.3_Pb_0.5_Sn_0.5_I_2.5_Br_0.5_ films show no sign of halide segregation during prolonged illumination.

## 2 Results and Discussion

### 2.1 Crystallization as Function of Antisolvent Dropping Time

**Figure 1:**
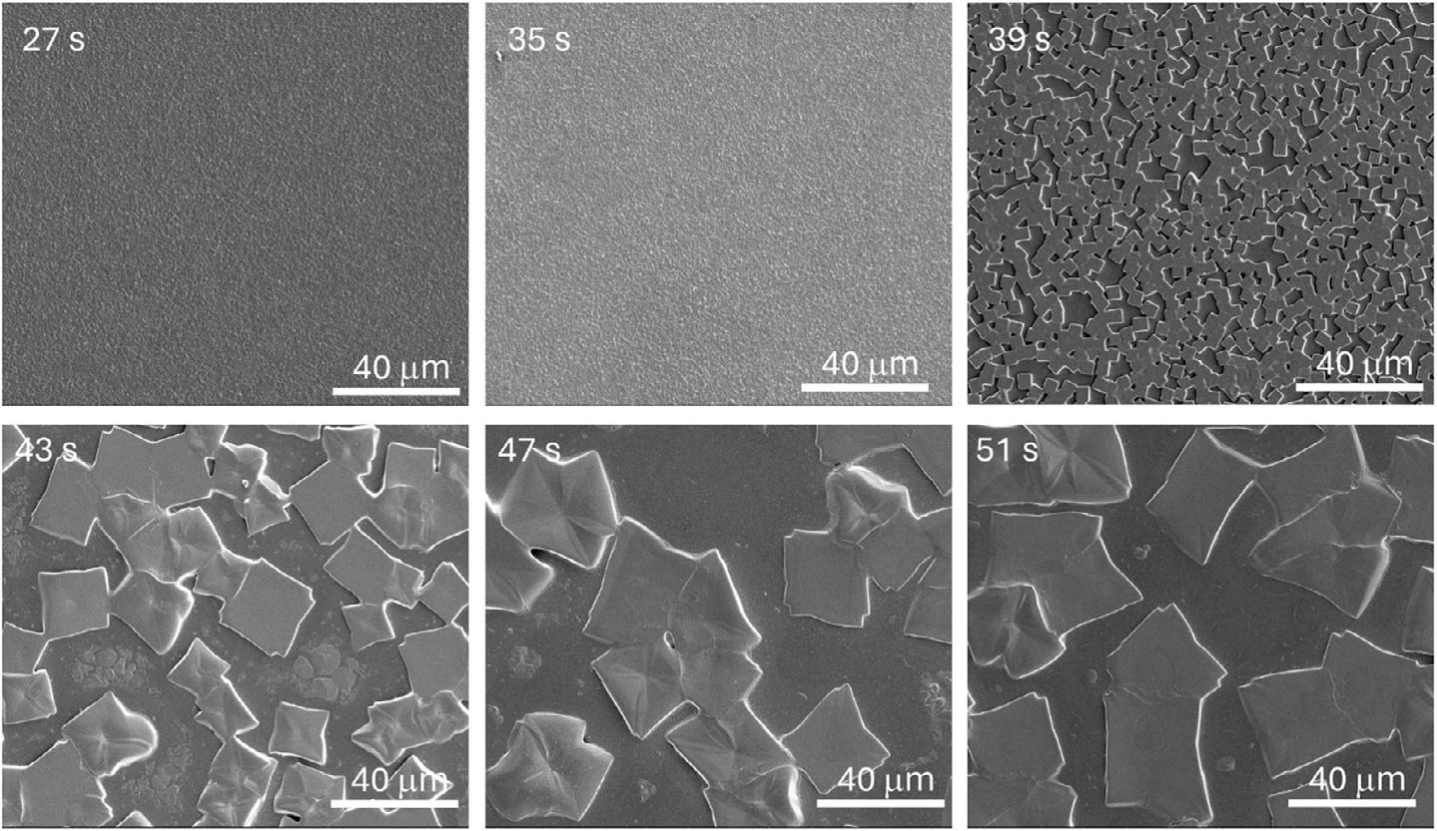
Scanning electron microscopy images of Cs_0.1_FA_0.6_MA_0.3_Pb_0.5_Sn_0.5_I_2.5_Br_0.5_ perovskite layers made with various antisolvent dropping delay times: Δ*t =* 27, 35, 39, 43, 47, and 51 s. The scale bar is 40 µm.

The starting point of our investigation is a narrow‐bandgap (1.26 eV) Cs_0.1_FA_0.6_MA_0.3_Pb_0.5_Sn_0.5_I_3_ perovskite, which we reported previously.^[^ ^15^ ^]^ To widen the bandgap, one‐sixth of iodide ions were replaced by bromide ions, resulting in a Cs_0.1_FA_0.6_MA_0.3_Pb_0.5_Sn_0.5_I_2.5_Br_0.5_ composition. This increases the bandgap from 1.24 to 1.34 eV (Figure S1, Supporting Information). Initially, the optimized deposition procedure for the full‐iodide perovskite^[^ ^15^ ^]^ was also used for the mixed‐halide perovskite. The spin‐coating process involved a stepwise acceleration to 4000 rpm, which was reached at Δ*t* = 24 s after the start (see Experimental Section for details). An antisolvent was used to effectively remove the precursor solvent and initiate crystallization. The type of antisolvent often influences the handling and timing of its deposition, to limit this, chlorobenzene (CB) was used because of its robustness to differences in the timing of the antisolvent application.^[^ ^16^ ^]^ After spin‐coating, a two‐step thermal annealing at 65 and 100 °C was employed. For the full‐iodide Pb‐Sn perovskite the optimum antisolvent dripping time was at Δ*t* = 51 s.^[^ ^15^ ^]^ However, for the bromide‐containing Pb‐Sn perovskite, this led to large individual crystals of ≈30–40 µm (**Figure** **1**; Figure S2, Supporting Information). These larger crystallites are reminiscent of film morphologies observed when perovskites are prepared from *N*,*N*‐dimethylformamide (DMF) without using an antisolvent as shown for, e.g., MAPbI_3_,^[^ ^17^ ^]^ FASnI_3_,^[^ ^18^ ^]^ and MAPbBr_3_.^[^ ^19^ ^]^ For MAPbBr_3_ perovskites, this has been attributed to its slower drying and retarded nucleation,^[^ ^20^ ^]^ or the formation of a crystalline intermediate.^[^ ^21^ ^]^ In the antisolvent procedure, the timing of dropping the antisolvent on the drying and spinning film is pivotal for the morphology of the resulting layer.^[^ ^20^, ^21^, ^22^, ^23^, ^24^ ^]^ When the antisolvent was deposited at Δ*t* = 51 s, the resulting films were similar to those made without antisolvent (Figure S3, Supporting Information). Next, the delay time was gradually decreased in steps of 4 s to Δ*t* = 27 s and films were analyzed using scanning electron microscopy (SEM). When Δ*t* was reduced from 51 to 39 s, the size of the perovskite crystals reduced considerably (Figure 1), going from a partly‐covered and translucent film to a more homogeneously‐covered and closed layer (Figures S4–S6, Supporting Information). When Δ*t* was reduced further to 35 and 27 s, closed, smooth, and visibly opaque layers were obtained with grain sizes of ≈1 µm. The transition point was around Δ*t* = 39 s, at which different locations on the film showed individual crystals with a range of sizes, next to regions where the separated crystals merged into closed areas with grains of 2−3 µm (Figure S6, Supporting Information). The different regions could also be observed with the naked eye.

The different layers showed X‐ray diffraction (XRD) peaks at 14.2°, 20.1°, 24.6°, 28.5°, 32.0°, and 35.1° corresponding to the (100), (110), (111), (200), (210), and (211) orientations for a cubic unit cell, with the highest intensities for the (*h*00) orientations (Figure S7, Supporting Information). The highest peak intensities were obtained for films prepared with the longest delay between the start of spinning and deposition of the antisolvent, where large crystallites are formed. Additional minor diffraction peaks were observed for Δ*t* ≥ 39 s, but these could not be unambiguously assigned.

**Figure 2:**
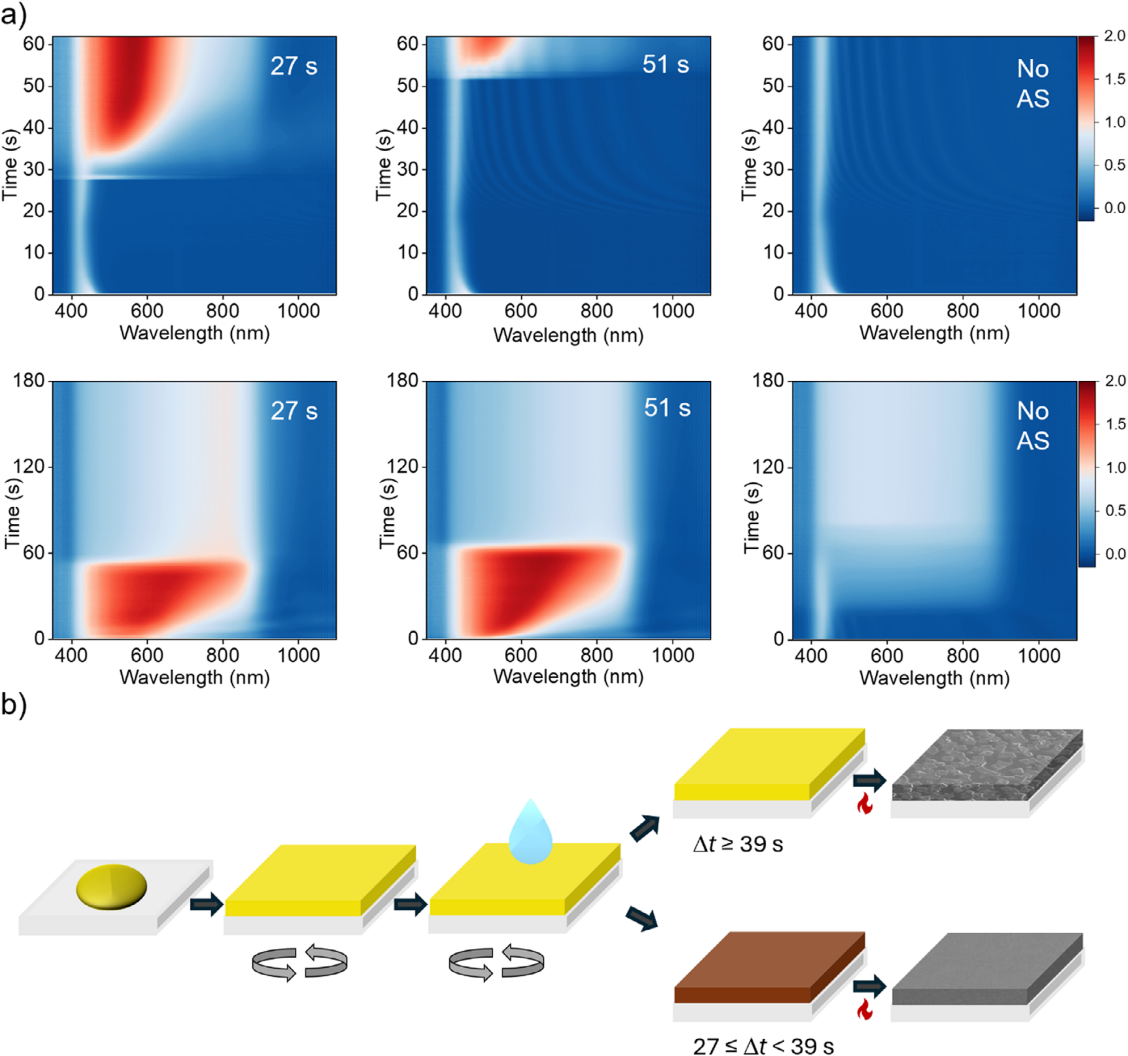
a) In situ absorption measurements during spin‐coating (top row) and thermal annealing at 65 °C (bottom row) for Δ*t* = 27, 51 s and without antisolvent. The heat maps show the absorbance, −^10^log(*I*_s_/*I*_b_), where *I*_s_ and *I*_b_ represent the intensities of the light reflected from the sample (*I*_s_) and from the blank (*I*_b_) substrate, each after subtracting spurious background intensity. b) Schematic of the spin‐coating procedure for Cs_0.1_FA_0.6_MA_0.3_Pb_0.5_Sn_0.5_I_2.5_Br_0.5_ perovskite layers. Late antisolvent dropping (Δ*t* ≥ 39 s) results in a yellow film after spin‐coating and a porous layer with large cubic crystals, while earlier application of the antisolvent (Δ*t* ≤ 35 s) induces crystallization to form a brown film during spin‐coating and eventually a closed layer after thermal annealing.

To gain further insight into the specific role of the antisolvent, the time delay before application of the antisolvent, and the function of thermal annealing during film formation, in situ optical absorption measurements were performed on films during spin‐coating and thermal annealing. **Figure** **2** shows the absorbance (i.e., optical density, −^10^log *A*) of films made with antisolvent deposition at Δ*t* = 27 and 51 s, and for a film prepared without antisolvent. Absorption spectra were recorded by using a fiber‐coupled spectrometer that measured specular and diffuse light that is reflected from the perovskite precursor film on a glass substrate that was covered on the rear‐side with an opaque white scattering layer.^[^ ^25^, ^26^ ^]^ Light reflected from the top surface of the drying film and from the film‐glass interface leads to interference patterns in the absorbance spectra. This is most prominent when the drying film has a uniform thickness, and hence most visible when it is still in a liquid (or wet) phase. The peaks and valleys of interference pattern shifts to shorter wavelengths when the film thickness reduces over time. The corresponding absorbance spectra versus time are shown in Figure S8 (Supporting Information).

Figure 2 shows that at the start of the measurement, just after the precursor solution is deposited onto the substrate, a sharp peak appears at ≈420 nm, which corresponds to the precursor complex in solution. During spin‐coating, the peak at 420 nm initially decreases during a flow phase in which excess solution is ejected off the substrate. Ejection of excess precursor solution leads to less absorption of the incoming light due to reducing film thickness and thus results in more reflection. When no antisolvent was used, this peak intensity reduced slightly due to the reduction in film thickness, and the peak position remained present until the end of spinning, leading to a yellow layer. Bruening and Tassone ascribed this yellow phase to the formation of an intermediate phase of precursor complexes sandwiching the solvent.^[^ ^21^ ^]^ The absence of a clear red‐shift indicates that no perovskite phase is formed. When an antisolvent was used, the initial peak immediately broadened to both the visible and near‐infrared regions, which we attribute to the formation of a narrow‐bandgap perovskite. For the Δ*t* = 27 s sample, an instant color transition from yellow to brown was observed upon the addition of the antisolvent. At longer spinning times, the film darkened accompanied by a noticeable red‐shift in the in situ absorption spectra, indicating the formation of a perovskite phase with a bandgap of ≈1.34 eV. For samples with Δ*t* between 43 and 51 s, the brown color was only observed temporarily or not at all, and these samples all yielded a yellow layer at the end of spinning. While the in situ absorption data do show a red‐shift of the absorbance for Δ*t* = 51 s, the slope of the absorbance at the long‐wavelength onset is very shallow, suggesting only partial formation of perovskite. In general, the absolute absorbance at the end of spinning is lower for these longer delay times.

After spin‐coating, the samples were transferred to a hotplate at 65 °C, and the absorbance was measured over time from the reflected light. The brown layers that were obtained from early antisolvent deposition turned black almost instantly, reaching their full absorbance. For the sample processed without antisolvent, the perovskite phase only appeared after ≈20 s of thermal annealing, followed by a slow proportional increase of the absorbance over the entire spectral range. Hence, thermal energy is needed to release solvent molecules and allow the formation of the perovskite phase.^[^ ^21^ ^]^ For the sample with Δ*t* = 51 s, the slope at the onset of absorbance increased upon annealing, indicating the formation of a more distinct perovskite phase. This demonstrates that thermal annealing is essential for the complete formation of the perovskite. For all measured samples, the films became hazy ≈40–50 s after converting to the perovskite phase, due to an increased surface roughness that leads to increased scattering and thus a lower absorbance. This roughening is most likely stemming from the evaporation of the remaining dimethyl sulfoxide (DMSO) or other residues. After ≈60 s, no further changes were detected, and all layers reached the same bandgap independent of Δ*t*, suggesting that the perovskites formed have similar stoichiometries. X‐ray photoelectron spectroscopy (XPS) was performed on perovskite layers prepared with antisolvent dropping at Δ*t* = 27, 39, and 51 s (Figure S9, Supporting Information). The elemental composition for layers with Δ*t* = 27 and 39 s is very similar, except for a small amount of Si in the Δ*t* = 39 s sample, consistent with the incomplete perovskite layer (Figure 1). In both layers, slight preferences were observed for iodide and lead at the top of the layer and tin and bromide at the bottom (Figure S9a, Supporting Information). Due to the limited surface coverage of the Δ*t* = 51 s sample by perovskite, the XPS showed a significant signal of Si (Figure S9b, Supporting Information).

The morphology of these layers is largely influenced by the delay time Δ*t* as seen from the SEM images in Figure 1. The observable brown phase for Δ*t* = 27 s is a clear indication of a perovskite phase that is formed by the antisolvent, which displaces the DMF/DMSO solvent mixture and creates a rapid oversaturation of the dissolved components, resulting in uniform nucleation. When the antisolvent is dropped after a significant delay, this brown phase is omitted, and the nucleation density is reduced. Only upon annealing the growth of the perovskite is stimulated due to the evaporation of the solvent, resulting in the large singular cubic crystals. Resulting from these experiments, further films were made with an antisolvent deposition delay of Δ*t* = 27 s since this resulted in smooth and closed layers that are most suitable for solar cell devices.

### 2.2 Effect of Thiocyanates on Crystallization

In the previous section, we demonstrated how processing parameters influence the crystallization behavior of the perovskite layer. However, crystallization is also affected by additives in the precursor solution. Thiocyanates (SCN^−^) are known to not only alter the crystallization but also passivate perovskites^[^ ^27^, ^28^ ^]^ by interacting with undercoordinated Sn^2+^ or Pb^2+^, therewith reducing defects,^[^ ^29^ ^]^ while the corresponding counterion can reduce A‐site vacancies in the bulk or at grain boundaries.

**Figure 3:**
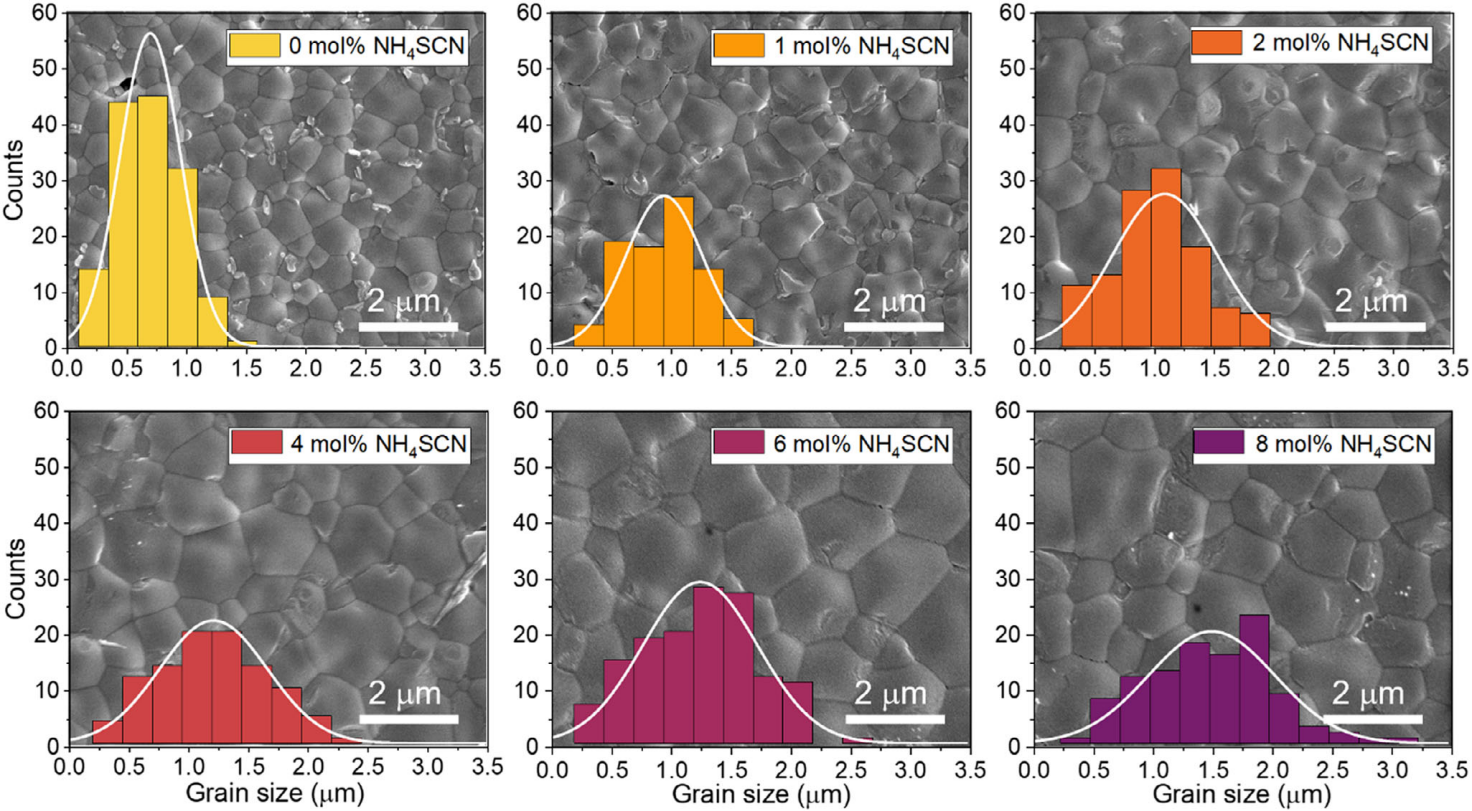
Statistical distribution of grain sizes overlaying scanning electron microscopy images of Cs_0.1_FA_0.6_MA_0.3_Pb_0.5_Sn_0.5_I_2.5_Br_0.5_ perovskite films made with 0, 1, 2, 4, 6, and 8 mol% NH_4_SCN.

The effect of ammonium thiocyanate (NH_4_SCN) was tested by adding 1, 2, 4, 6, and 8 mol% to the precursor solution. SEM images show a large increase in grain size from an average grain size of 700 nm for a film without additives to 1500 nm for the sample with 8 mol% NH_4_SCN (**Figure** **3**; Table S2, Supporting Information), while the thickness of the perovskite film remained at ≈840 nm (Table S3, Supporting Information). All concentrations tested resulted in films with a Gaussian crystal size distribution and provided pinhole‐free layers. Despite the increase in grain size, the crystal orientation and intensity of the XRD peaks were identical for all samples (Figure S10, Supporting Information). The samples exhibited clear XRD peaks for the (100) and (200) orientations at ≈14.2° and 28.6°, and minor peaks at 20.2°, 24.7°, 32.1°, and 35.2° related to the (110), (111), (210), and (211) orientations. Overall, a relatively low intensity PbI_2_ peak was observed at 12.8°, indicating a correct stoichiometry in solution and during film fabrication.

XPS depth‐profiling performed on the perovskite layers processed with 0 and 6 mol% NH_4_SCN (Figure S11, Supporting Information) showed identical elemental depth profiles for Br, I, Pb, Sn, Cs, and N independent of the use of the additive. In situ absorption measurements during spin‐coating, with and without the use of NH_4_SCN, did not reveal significant differences in the growth of the perovskite phase with and without additive (Figure S12, Supporting Information). During annealing, however, the switch to a hazy film was retarded with 5 s for the layer with NH_4_SCN. The delay in the evaporation of DMSO or other residues could be caused by the increase in grain size and thus reduced grain boundaries. XPS surface analysis and depth profiling revealed no clearly distinguishable S 2p signals, indicating the absence of a detectable amount of SCN^−^ in the final film. This is attributed to the volatility of NH_4_SCN during thermal annealing in accordance with previous observations.^[^ ^30^ ^]^

**Figure 4:**
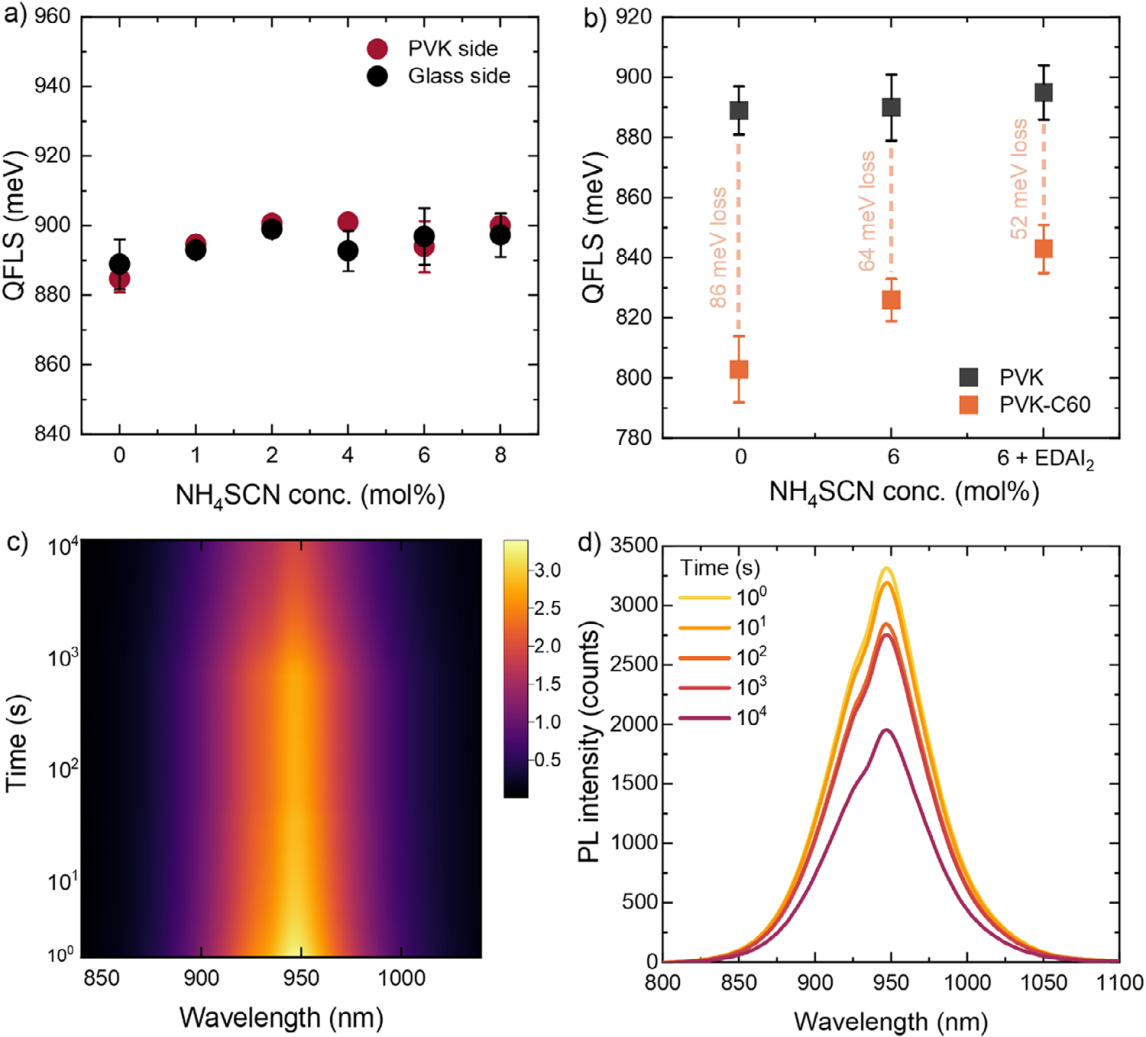
a) The quasi‐Fermi level splitting (QFLS) of Cs_0.1_FA_0.6_MA_0.3_Pb_0.5_Sn_0.5_I_2.5_Br_0.5_ perovskite films processed with 0, 1, 2, 4, 6, or 8 mol% NH_4_SCN. The absolute photoluminescence was measured from both the glass and perovskite (PVK) sides. b) QFLS of the perovskite layer without and with C_60_ on top measured through the glass side. The three samples represent the perovskite without bulk additive, with 6 mol% NH_4_SCN, and with 6 mol% NH_4_SCN plus top passivation with EDAI_2_. c) and d) Photoluminescence over time during continuous 405 nm illumination at 1‐sun equivalent intensity of the perovskite layer, processed without additives.

To determine the influence of NH_4_SCN on the optoelectronic properties of the perovskite film, the absolute photoluminescence photon flux was measured from both the glass and the perovskite side, which enables one to determine the quasi‐Fermi level splitting (QFLS) from Planck's radiation law (**Figure** **4**a). The detailed‐balance limit for the 1.34 eV bandgap perovskite is at ≈1080 meV, and any deviation from that signifies the losses due to non‐radiative recombination. When measuring from the perovskite side, the layer processed without NH_4_SCN had a QFLS of 885 meV, corresponding to 82% of the detailed‐balance limit. The QFLS increases slightly when adding 1 to 2 mol% NH_4_SCN after which it remains more constant (Figure 4a). The small 10–15 meV gain is close to the standard deviation of the experiment and must be interpreted with caution. The grain size shows an analogous behavior (Table S2, Supporting Information), also changing most with small amounts of NH_4_SCN. Hence, the passivating effects of NH_4_SCN for this perovskite composition are small and are possibly related to the reduction of grain boundaries when the grain size increases. The QFLS was also measured for a partial device stack in which a C_60_ layer is evaporated on top of the perovskite layer (Figure 4b). Without NH_4_SCN, C_60_ causes an additional loss of 86 meV compared to the neat perovskite layer. This loss is well‐documented,^[^ ^31^, ^32^, ^33^ ^]^ and attributed to the formation of interfacial trap states at the perovskite/C_60_ interface. With 6 mol% NH_4_SCN the loss in QFLS decreased to 64 meV when C_60_ was added on top. This indicates that the NH_4_SCN additive reduces the non‐radiative losses. Because no SCN^−^ can be detected at the top surface, it seems improbable that a surface layer of NH_4_SCN causes the passivating effect. As a tentative explanation we propose that the larger grains of perovskite films processed with NH_4_SCN create a smaller interfacial area with C_60_ compared to films with smaller grains. Next, a commonly used surface passivator for lead‐tin perovskites, EDAI_2_, was used to further reduce the non‐radiative recombination losses to 52 meV. Similarly to the NH_4_SCN, EDAI_2_ did not passivate the perovskite surface itself, but further improved the interface with the C_60_ layer. The stabilizing effects of diammonium molecules has been ascribed to a field‐effect that reduces recombination between holes in the perovskite and electrons in the C_60_ layer,^[^ ^34^, ^35^, ^36^ ^]^ and to increasing the spatial separation between the perovskite semiconductor and the C_60_ layer.^[^ ^33^ ^]^

Light‐induced halide segregation is commonly observed in lead‐based mixed‐halide perovskites and severely affects the stability. To investigate light‐induced halide segregation in the mixed‐metal mixed‐halide perovskites, Cs_0.1_FA_0.6_MA_0.3_Pb_0.5_Sn_0.5_I_2.5_Br_0.5_ films on glass were continuously illuminated at 530 nm with a 1‐sun equivalent intensity for ≈3 h. The photoluminescence spectra recorded versus time of samples processed without NH_4_SCN are shown in Figure 4c. No new peaks or peak‐shifts were observed, indicating that light‐induced halide segregation does not occur for the Cs_0.1_FA_0.6_MA_0.3_Pb_0.5_Sn_0.5_I_2.5_Br_0.5_ perovskite. The photoluminescence intensity gradually drops by ≈40% after 10^4^ s of illumination (Figure 4d). There is no initial increase of photoluminescence intensity, a phenomenon that is often observed and ascribed to a dynamic process in which the perovskite undergoes changes that reduce the number of traps or annihilate defects that improve its optoelectronic properties.^[^ ^37^, ^38^ ^]^ The observed drop of photoluminescence intensity during prolonged continuous illumination is common for perovskites under inert conditions.^[^ ^39^, ^40^, ^41^, ^42^ ^]^ Explanations that have been proposed are ion migration that results in the formation of defect traps^[^ ^41^, ^43^ ^]^ or the formation and release of iodine due to halide interstitials and lead vacancies.^[^ ^42^, ^44^ ^]^ The QFLS remained similar throughout the measurement with only a small drop of max. 3 meV. Essentially identical results have been obtained for the Cs_0.1_FA_0.6_MA_0.3_Pb_0.5_Sn_0.5_I_2.5_Br_0.5_ perovskite when processed with 6 mol% NH_4_SCN, and with NH_4_SCN in combination with EDAI_2_ surface passivation (Figure S13, Supporting Information). No halide segregation is induced when measuring the photoluminescence at 1‐sun equivalent intensity for ≈3 h. The photoluminescence spectra, however, show a small shoulder at lower wavelengths. This shoulder is present for all concentrations of NH_4_SCN used, independent of the excitation wavelength, and independent of excitation intensity (Figure S14, Supporting Information). Such non‐Gaussian peaks may arise from several factors, including the presence of defect states, halide segregation, and structural heterogeneity. The spectral shape remains unaltered for almost 3 h of illumination at 1‐sun equivalent intensity. In our view, this excludes halide segregation and defects as the primary cause, because both are expected to evolve over time. Although we cannot exclude structural heterogeneity, we propose that the non‐Gaussian photoluminescence line shape originates from self‐absorption and thin‐film cavity effects as explained previously,^[^ ^45^ ^]^ and encountered in several studies.^[^ ^46^, ^47^, ^48^, ^49^ ^]^

### 2.3 Photovoltaic Devices

The photovoltaic performance of the Cs_0.1_FA_0.6_MA_0.3_Pb_0.5_Sn_0.5_I_2.5_Br_0.5_ perovskite was tested using a p‐i‐n configuration with a glass substrate, an indium tin oxide (ITO) front electrode, and a poly(3,4‐ethylenedioxythiophene):poly(styrene sulfonate) (PEDOT:PSS) hole transport layer (HTL). The electron transport layer (ETL) consisted of a 20 nm C_60_ and 8 nm bathocuproine (BCP) bi‐layer, and the back electrode was a 100 nm layer of silver. Devices were made with 0, 1, 2, 4, 6, and 8 mol% NH_4_SCN in the perovskite precursor solution. The performance of record devices did not necessarily increase with the addition of NH_4_SCN but higher concentrations increased the reproducibility of the devices and reduced the hysteresis between the forward and reverse sweeps (Figure S15 and Table S4, Supporting Information). The external quantum efficiency (EQE) also showed a smaller dependence for the samples with NH_4_SCN when recorded with or without bias light (Figure S16, Supporting Information).

**Figure 5:**
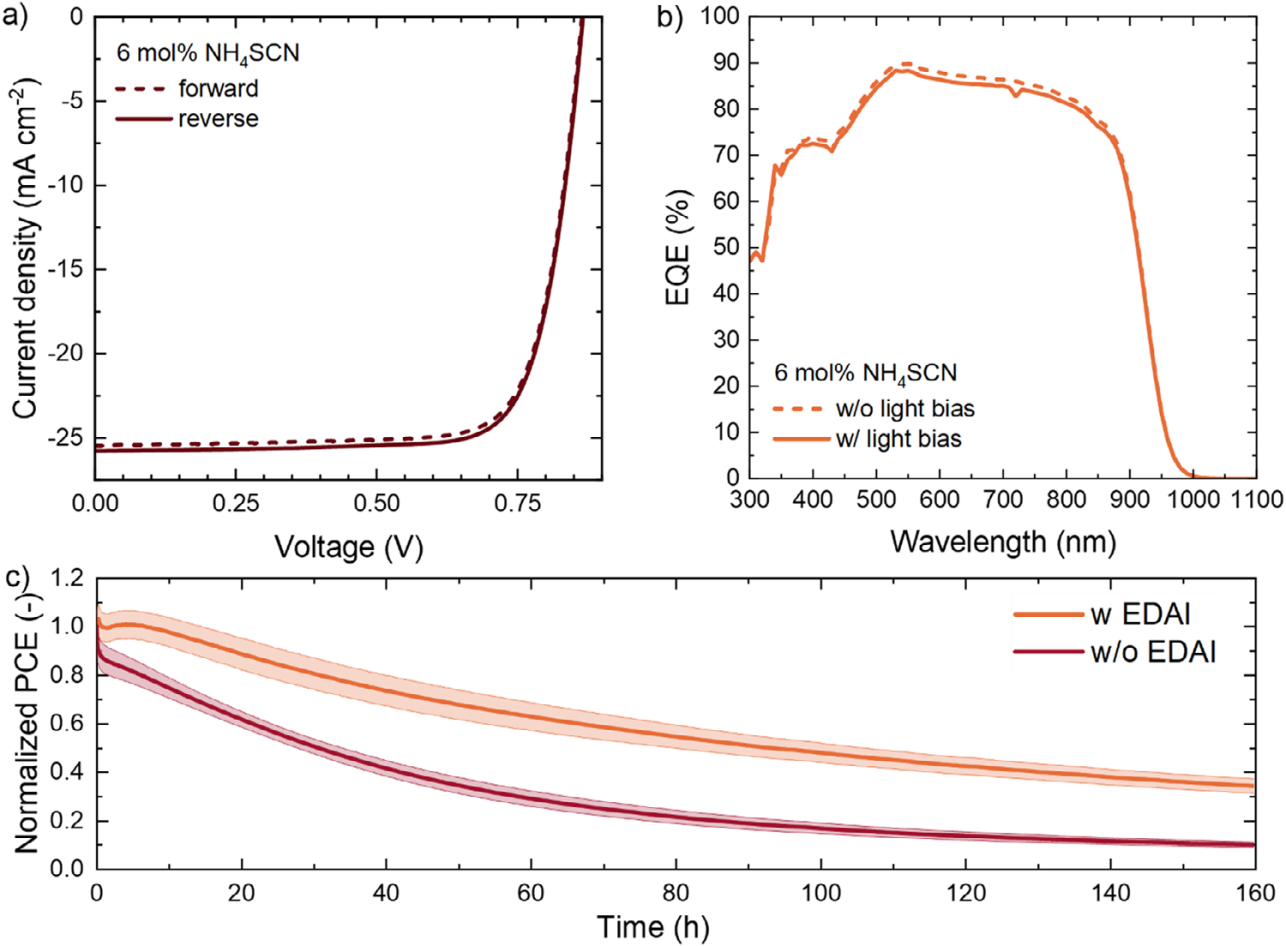
a) Current density‐voltage characteristics scanned in forward (dashed) and reverse (solid) directions for the champion Cs_0.1_FA_0.6_MA_0.3_Pb_0.5_Sn_0.5_I_2.5_Br_0.5_ perovskite solar cell processed with 6 mol% NH_4_SCN, EDAI_2_ interface passivation, a thin PEDOT:PSS HTL, and an antireflective coating. b) External quantum efficiency of the same device, recorded without and with 1‐sun equivalent 530 nm light bias. c) MPP tracking of the solar cells processed with and without surface top (EDAI_2_) passivation during 160 h of continuous operation under continuous white‐light LED illumination at 1‐sun equivalent intensity. The data represent the mean standard deviation calculated from 4 or 6 independent devices.

The solar cell processed with 6 mol% NH_4_SCN was further optimized using EDAI_2_, which is a commonly used top‐surface passivator for Pb‐Sn perovskites.^[^ ^6^, ^50^, ^51^ ^]^ Applying EDAI_2_ in a concentration of 0.25 mg mL^−1^ dissolved in a 2:1 isopropanol‐chlorobenzene mixture led to a significant improvement in the *V*_OC_ from 0.80 to 0.85–0.87 V, indicating a reduction of non‐radiative losses at the perovskite‐C_60_ interface. Other interface passivators such as 1,3‐propanediammonium diiodide (PDAI_2_) and choline chloride (ChCl) were also tested because they are successful for full‐lead‐based iodide and mixed‐halide perovskites.^[^ ^52^, ^53^, ^54^, ^55^ ^]^ However, no increase in *V*_OC_ was seen in combination with the Cs_0.1_FA_0.6_MA_0.3_Pb_0.5_Sn_0.5_I_2.5_Br_0.5_ perovskite. The *J*_SC_ was further improved by diluting the PEDOT:PSS dispersion used for the HTL with 1‐propanol in a 1:2 ratio to reduce parasitic absorption and by applying an antireflection coating (ARC) to enhance absorption. The EQE‐integrated *J*_SC,EQE_ of the optimized device was 28.4 mA cm^−2^. Together with a FF of 0.77, a PCE_EQE_ of 19.0% was obtained (**Figure** **5** and **Table** **1**). The second derivative of the EQE spectrum confirms the bandgap of the perovskite to be at 1.34 eV (Figure S17, Supporting Information). Maximum power point (MPP) tracking under illumination at 1‐sun equivalent intensity was performed for 160 h for four to six nominally identical devices. The stability under operating conditions of the optimized Cs_0.1_FA_0.6_MA_0.3_Pb_0.5_Sn_0.5_I_2.5_Br_0.5_ solar cells is moderate (*T*_80_ = 30.5 h) and positively affected by the use of EDAI_2_ for top‐passivation.

**Table 1:** Photovoltaic parameters of the optimized device with 6 mol% NH_4_SCN.

	*J*_SC_ [mA cm^−2^]	*V*_OC_ [V]	FF [‐]	PCE [%]	*J*_SC,EQE_ [mA cm^−2^]	PCE_EQE_ [%]
Reverse	25.8	0.87	0.77	17.3	28.4	19.0
Forward	25.4	0.86	0.77	16.8	28.4	18.8

## 3 Conclusion

In this work, we show that the incorporation of one‐sixth of bromide into full‐iodide mixed lead‐tin perovskites allows for the formation of a 1.34 eV bandgap Cs_0.1_FA_0.6_MA_0.3_Pb_0.5_Sn_0.5_I_2.5_Br_0.5_ perovskite. Introducing bromide increases the crystallization rate resulting in singular cubic crystals of ≈40 µm. The use of an antisolvent and the timing of deposition during the spin‐coating process was found to be crucial for obtaining the desired smooth and pinhole‐free film morphology. An early antisolvent‐deposition time of 27 s after starting spin‐coating of the precursor allowed for the formation of uniform and closed layers with a thickness of 840 nm. The addition of few mole percent of NH_4_SCN to the precursor solution increased the grain size and reduced non‐radiative recombination losses at the perovskite‐C_60_ interface. NH_4_SCN also reduced the hysteresis in current density – voltage scans and improved reproducibility and stability under operating conditions. The optimum NH_4_SCN concentration was 6 mol%. The performance was further improved using EDAI_2_ to passivate the perovskite‐C_60_ interface and reduce non‐radiative recombination losses. A champion device with a *V*_OC_ of 0.87 V, *J*_SC_ of 28.4 mA cm^−2^, FF of 0.77, and PCE of 19.0% was obtained. This PCE is among the highest obtained for the 1.3 to 1.4 eV bandgap range (Table S5, Supporting Information), and significantly higher than obtained so far for mixed‐metal mixed‐halide perovskites. The results give credence to suggest that complex triple‐cation, dual‐metal, dual‐halide perovskite compositions deserve more attention than they have received until now and develop strategies that further reduce non‐radiative recombination and enhance stability for these materials.

## 4 Experimental Section

### Materials

All materials were used as received without further purification and stored under nitrogen. Lead(II) iodide (PbI_2_, 99.99% trace metal basis), and lead(II) bromide (PbBr_2_, >98.0%) were purchased from TCI Chemicals. Anhydrous tin(II) iodide beads (SnI_2_, 99.99% trace metals basis), tin(II) fluoride (SnF_2_, 99%), anhydrous cesium iodide beads (CsI, 99.999% trace metals basis), ammonium thiocyanate (ACS reagent, ≥97.5%), anhydrous *N,N*‐dimethylformamide (DMF, 99.8%), anhydrous dimethyl sulfoxide (DMSO, ≥99.9%), anhydrous chlorobenzene (CB, 99.8%), and anhydrous isopropanol (IPA, 99.5%) were purchased from Sigma–Aldrich. Methylammonium iodide (MAI, >99.99%), formamidinium iodide (FAI, >99.99%), and 1,2‐ethylenediammonium diiodide (EDAI_2_) were purchased from Greatcell Solar Materials. Poly(3,4‐ethylenedioxythiophene):poly(styrene sulfonate) (PEDOT:PSS, PVP Al 4083) was purchased from Heraeus Clevios. Bathocuproine (BCP, >99.5%) was purchased from Lumtec, and C_60_ fullerene (99.99%) from SES Research.

### Solution Preparation

All solutions were prepared in a nitrogen environment. The perovskite precursor solution for a perovskite film with a nominal composition of Cs_0.1_FA_0.6_MA_0.3_Pb_0.5_Sn_0.5_I_2.5_Br_0.5_ was made by dissolving 0.45 m PbI_2_, 0.45 m PbBr_2_, 0.9 m SnI_2_, 0.18 m CsI, 1.08 m FAI, and 0.54 m MAI in a DMF/DMSO mixture with a volume ratio of 3:1. Additionally, 0.045 m SnF_2_ was added. For the devices with different NH_4_SCN concentrations, separate precursor solutions were made with either 0, 1, 2, 4, 6, or 8 mol% of NH_4_SCN relative to the total metal (PbI_2_, PbBr_2_, and SnI_2_) concentration. The solutions were stirred for 40 min at 45 °C. After cooling to room temperature, the solutions were filtered using a 0.22 µm PTFE syringe filter. For the EDAI_2_ solution, 0.25 mg mL^−1^ was dissolved in a IPA/CB mixture with a volume ratio of 2:1. First, the EDAI_2_ was dissolved in the IPA only and stirred at 60–70 °C for ≈2 h, after which the CB was added to stir for another 10 min at the elevated temperature. After cooling to room temperature, the solution was filtered using a 0.22 µm PTFE syringe filter.

### Perovskite Film Fabrication

The perovskite film was spin‐coated on glass using a volume of 200 µL. The spin‐coating procedure consisted of two steps. First (Δ*t* = 0) the substrate was accelerated at 100 rpm s^−1^ to 1000 rpm. After 10 s at 1000 rpm (Δ*t =* 20 s), using an acceleration of 750 rpm s^−1^, the spin speed was increased to 4000 rpm (Δ*t* = 24 s) and kept constant for 40 s. The antisolvent (400 µL of chlorobenzene) was applied at Δ*t* = 27, 31, 35, 39, 43, 47, or 51 s after starting the spinning. All films were annealed at 65 °C for 10 min followed by 10 min at 100 °C.

### Device Fabrication

For the full devices, patterned‐ITO glass substrates were sonicated in acetone, scrubbed clean in soapy water, sonicated in soapy water, flushed with demi‐water, and sonicated in isopropanol. The substrates had a 30 min UV‐ozone treatment, after which they were immediately used for PEDOT:PSS spin‐coating in ambient air. The aqueous dispersion was filtered using a 0.45 µm PVDF filter, and 150 µL was deposited onto the substrate. The spin‐coating of PEDOT:PSS consisted of two steps: first, the substrate spun at 500 rpm for 10 s using an acceleration of 20 000 rpm s^−1^, second, the substrate spun at 4000 rpm for 30 s with an acceleration of 20 000 rpm s^−1^. The films were first annealed in ambient air for 20 min at 140 °C after which they were transferred to the N_2_ glovebox, where they were further annealed for 30 min at 140 °C. After cooling to room temperature, the perovskite film was spin‐coated with the procedure described above. The EDAI_2_ top passivator was dynamically spin‐coated at 4000 rpm for 30 s using an acceleration of 1333 rpm s^−1^ and directly annealed for 5 min at 100 °C. C_60_ (20 nm) and BCP (8 nm) were thermally evaporated at a rate of 0.2 Å s^−1^ under high vacuum (2–6 × 10^−7^ mbar). The Ag top electrode (100 nm) was thermally evaporated using an initial rate of 1 Å s^−1^ increasing to 3 Å s^−1^. An anti‐reflective coating was used for the devices with 6 mol% NH_4_SCN and EDAI_2_ (Figure 5 and Table 1).

### Photovoltaic Performance Characterization

The *J−V* and EQE measurements were taken under nitrogen atmosphere (<1 ppm O_2_ and <1 ppm H_2_O). For the *J−V* measurements, a tungsten‐halogen lamp (100 mW cm^2^) was used with a daylight filter (Hoya LB120) and UV filter (Schott GG385) to simulate the AM1.5G solar spectrum. A shadow mask with 0.0676 and 0.1296 cm^2^ aperture sizes was used to define the illuminated active area. The current was measured over an applied voltage range of −0.5 to +1.0 V (forward sweep) or in the reverse direction (backward sweep) applied with a Keithley 2400 source measure unit using a step size of 0.01 V and a scan rate of 0.25 V s^−1^. The same set‐up was used for the stability test in which the voltage kept constant and the current was measured over time. For the EQE measurements, the light of a 50 W tungsten halogen lamp was chopped at 158 Hz before passing into a monochromator (Oriel, Cornerstone 130). The signal of the solar cell was amplified using a pre‐amplifier (Stanford Research, SR 570) and measured with a lock‐in amplifier (Stanford Research, SR 830). A green LED (Thorlabs, M530L3) was used as a light bias to generate 1‐sun equivalent illumination intensity. The spectral response was integrated over the AM1.5G spectrum using a reference silicon cell to yield in the *J*_SC,EQE_. Due to a known mismatch between the irradiance of the light source used for the *J−V* measurements and the AM1.5G spectrum, the *J*_SC_ is ≈10% less than *J*_SC,EQE_, which is considered as the more accurate estimate. Stability tests of solar cells were performed by maximum power point tracking for 160 h of four to six nominally identical solar cells that were kept at 25 °C under a stream of N_2_ and continuously illuminated at 1‐sun equivalent intensity with white‐light LEDs (Automatic Research GmbH).

### Film Characterization

Scanning electron microscopy (SEM) images were taken by a FEI Quanta 3D FEG microscope using a 5 keV electron beam and secondary electron detector. Au or Pt was sputtered on the samples before imaging. Surface profilometry was performed with a Veeco Dektak 150. X‐ray diffraction (XRD) was recorded using a Bruker 2D phaser (Cu Kα radiation, *λ* = 1.5406 Å). Measurements were performed in the range of 10°−40° with a step size of 0.01° and collection time of 0.2 s. A divergence slit of 0.6 mm and an anti‐scatter screen of 0.5 mm were employed. For the samples with NH_4_SCN, a step size of 0.05° and collection time of 0.5 s.

### X‐Ray Photoelectron Spectroscopy

XPS measurements were performed using a Thermo‐Scientific K‐Alpha with a 180° double‐focusing hemispherical analyzer and a 128‐channel detector. Monochromatic Al Kα (1486.6 eV) radiation was used, and the X‐ray spot size was 400 µm. For the surface analysis, a survey spectrum was first measured for 12 scans (dwell time of 20 ms) with a pass energy of 200 eV. High‐resolution scans (30 times, dwell time of 50 ms) of each element were conducted with a pass energy of 50 eV. The depth‐profile measurement was performed in etching mode with an ion energy of 1000 eV and low current (sputter rate estimate of 0.05 nm s−1). Each etch cycle had a duration of 45 s, and 110 total levels were measured.

### Quasi‐Fermi Level Splitting

The sample was illuminated using a 455 nm Thorlabs M455F3 fiber‐coupled LED. The light passes through an in‐fiber filter holder containing an Edmund Optics 550 nm short‐pass filter before entering an integrating sphere (Avantes AvaSphere‐30‐REFL). The PL emission from the sample was collected through an optical fiber mounted to the side of the sphere and was passed through an in‐fiber filter holder with an Edmund Optics 550 nm long‐pass filter before it was collected by a spectrometer (Avantes AvaSpec‐HSC1024X58TEC‐EVO). The setup was calibrated using an Avantes halogen lamp with known spectral irradiance.

### Halide Segregation

To measure halide segregation, samples were continuously illuminated for 3 h using a 405 nm Thorlabs M405FP1 fiber‐coupled LED. A custom‐built fiber holder positioned an in‐fiber filter holder containing an Edmund Optics 550 nm short‐pass filter. A second fiber, equipped with a 550 nm long‐pass filter (Edmund Optics), was connected to the same holder and directed the photoluminescence signal to a spectrometer (Avantes AvaSpec‐HSC1024X58TEC‐EVO). The photoluminescence signals were collected at logarithmically increasing time intervals.

### In Situ Absorption

In situ absorption spectra were captured by measuring in situ reflectance spectra of perovskite films, and by sequentially converting reflection to absorption. White spray paint was applied to the rear side of glass substrates to eradicate transmission and absorption losses that could stem from non‐perovskite related interference and absorption, so that one can assume that reflection and absorption are related via: Reflection = 1 – Absorption. The painted substrate was placed onto either a spin‐coater or hotplate in a N_2_‐filled atmosphere. A halogen light was then focused onto the center of the substrate, and the scattered light was collected at an off‐specular angle with an optical fiber connected to a spectrometer. The absorbance was then calculated as:

(1) $$A\left(\lambda ,t\right)=-log_{10}\left(\frac{I_{m}\left(\lambda ,t\right)-I_{m,dark}\left(\lambda \right)}{I_{m,blank}\left(\lambda \right)-I_{m,dark}\left(\lambda \right)}\right)$$

where *I_m_* (λ,*t*) is the time‐dependent measured photon count of the sample (absorbance), *I*_*m*,*dark*_(λ) represents the noise of the spectrometer, and *I*_*m*,*blank*_(λ) represents a measurement on a white substrate without perovskite, representing full reflection and thus zero absorption, as reference.

## Conflict of Interest

The authors declare no conflict of interest.

## Supporting information



Supporting Information

Supporting Information

## Data Availability

The data that support the findings of this study are available from the corresponding author upon reasonable request.

## References

[1] H. Chen , C. Liu , J. Xu , A. Maxwell , W. Zhou , Y. Yang , Q. Zhou , A. S. R. Bati , H. Wan , Z. Wang , L. Zeng , J. Wang , P. Serles , Y. Liu , S. Teale , Y. Liu , M. I. Saidaminov , M. Li , N. Rolston , S. Hoogland , T. Filleter , M. G. Kanatzidis , B. Chen , Z. Ning , E. H. Sargent , Science 2024, 384, 189. 38603485 10.1126/science.adm9474

[2] Z. Liang , Y. Zhang , H. Xu , W. Chen , B. Liu , J. Zhang , H. Zhang , Z. Wang , D. H. Kang , J. Zeng , X. Gao , Q. Wang , H. Hu , H. Zhou , X. Cai , X. Tian , P. Reiss , B. Xu , T. Kirchartz , Z. Xiao , S. Dai , N. G. Park , J. Ye , X. Pan , Nature 2023, 624, 557. 37913815 10.1038/s41586-023-06784-0 PMC10733143

[3] O. Almora , G. C. Bazan , C. I. Cabrera , L. A. Castriotta , S. Erten‐Ela , K. Forberich , K. Fukuda , F. Guo , J. Hauch , A. W. Y. Ho‐Baillie , T. J. Jacobsson , R. A. J. Janssen , T. Kirchartz , R. R. Lunt , X. Mathew , D. B. Mitzi , M. K. Nazeeruddin , J. Nelson , A. F. Nogueira , U. W. Paetzold , B. P. Rand , U. Rau , T. Someya , C. Sprau , L. Vaillant‐Roca , C. J. Brabec , Adv. Energy Mater. 2024, 14, 2303173.

[4] L. Wang , H. Bi , J. Liu , Y. Wei , Z. Zhang , M. Chen , A. K. Baranwal , G. Kapil , T. Kitamura , S. Yang , Q. Miao , Q. Shen , T. Ma , S. Hayase , ACS Energy Lett. 2024, 9, 6238.

[5] M. Aldamasy , Z. Iqbal , G. Li , J. Pascual , F. Alharthi , A. Abate , M. Li , Phys. Chem. Chem. Phys. 2021, 23, 23413. 34533139 10.1039/d1cp02596a

[6] S. Hu , K. Otsuka , R. Murdey , T. Nakamura , M. A. Truong , T. Yamada , T. Handa , K. Matsuda , K. Nakano , A. Sato , K. Marumoto , K. Tajima , Y. Kanemitsu , A. Wakamiya , Energy Environ. Sci. 2022, 15, 2096.

[7] J. Zhou , S. Fu , S. Zhou , L. Huang , C. Wang , H. Guan , D. Pu , H. Cui , C. Wang , T. Wang , W. Meng , G. Fang , W. Ke , Nat. Commun. 2024, 15, 2324. 38485961 10.1038/s41467-024-46679-w PMC10940575

[8] R. Lin , Y. Wang , Q. Lu , B. Tang , J. Li , H. Gao , Y. Gao , H. Li , C. Ding , J. Wen , P. Wu , C. Liu , S. Zhao , K. Xiao , Z. Liu , C. Ma , Y. Deng , L. Li , F. Fan , H. Tan , Nature 2023, 620, 994. 37290482 10.1038/s41586-023-06278-z

[9] Z. Liang , H. Xu , Y. Zhang , G. Liu , S. Chu , Y. Tao , X. Xu , S. Xu , L. Zhang , X. Chen , B. Xu , Z. Xiao , X. Pan , J. Ye , Adv. Mater. 2022, 34, 2110241. 10.1002/adma.202110241 35230736

[10] X. Lian , J. Chen , Y. Zhang , M. Qin , J. Li , S. Tian , W. Yang , X. Lu , G. Wu , H. Chen , Adv. Funct. Mater. 2019, 29, 1807024.

[11] N. Li , Z. Zhu , J. Li , A. K. Y. Jen , L. Wang , Adv. Energy Mater. 2018, 8, 1800525.

[12] S. Lee , D. W. Kang , ACS Appl. Mater. Interfaces 2017, 9, 22432. 28650647 10.1021/acsami.7b04011

[13] Z. Yang , A. Rajagopal , S. B. Jo , C. C. Chueh , S. Williams , C. C. Huang , J. K. Katahara , H. W. Hillhouse , A. K. Y. Jen , Nano Lett. 2016, 16, 7739. 27960463 10.1021/acs.nanolett.6b03857

[14] S. Lee , J. Moon , J. Ryu , B. Parida , S. Yoon , D. G. Lee , J. S. Cho , S. Hayase , D. W. Kang , Nano Energy 2020, 77, 105309.

[15] L. M. Kessels , W. H. M. Remmerswaal , L. M. van der Poll , L. Bellini , L. J. Bannenberg , M. M. Wienk , T. J. Savenije , R. A. J. Janssen , Sol. RRL 2024, 8, 2400506.

[16] A. D. Taylor , Q. Sun , K. P. Goetz , Q. An , T. Schramm , Y. Hofstetter , M. Litterst , F. Paulus , Y. Vaynzof , Nat. Commun. 2021, 12, 1878. 33767163 10.1038/s41467-021-22049-8 PMC7994557

[17] J. H. Im , I. H. Jang , N. Pellet , M. Grätzel , N. G. Park , Nat. Nanotechnol. 2014, 9, 927. 25173829 10.1038/nnano.2014.181

[18] S. J. Lee , S. S. Shin , Y. C. Kim , D. Kim , T. K. Ahn , J. H. Noh , J. Seo , S. Il Seok , J. Am. Chem. Soc. 2016, 138, 3974. 26960020 10.1021/jacs.6b00142

[19] J. H. Heo , D. H. Song , S. H. Im , Adv. Mater. 2014, 26, 8179. 25348285 10.1002/adma.201403140

[20] J. Kumar , R. Kumar , K. Frohna , D. Moghe , S. D. Stranks , M. Bag , Phys. Chem. Chem. Phys. 2020, 22, 26592. 33201960 10.1039/d0cp05467d

[21] K. Bruening , C. J. Tassone , J. Mater. Chem. A 2018, 6, 18865.

[22] R. Szostak , P. E. Marchezi , A. D. S. Marques , J. C. Da Silva , M. S. De Holanda , M. M. Soares , H. C. N. Tolentino , A. F. Nogueira , Sustain. Energy Fuels 2019, 3, 2287.

[23] T. Bin Song , Z. Yuan , F. Babbe , D. P. Nenon , E. Aydin , S. De Wolf , C. M. Sutter‐Fella , ACS Appl. Energy Mater. 2020, 3, 2386.

[24] K. Wang , M. C. Tang , H. X. Dang , R. Munir , D. Barrit , M. De Bastiani , E. Aydin , D. M. Smilgies , S. De Wolf , A. Amassian , Adv. Mater. 2019, 31, 1808357. 10.1002/adma.201808357 31206857

[25] J. J. Van Franeker , M. Turbiez , W. Li , M. M. Wienk , R. A. J. Janssen , Nat. Commun. 2015, 6, 6229. 25656313 10.1038/ncomms7229

[26] J. Wang , K. Datta , J. Li , M. A. Verheijen , D. Zhang , M. M. Wienk , R. A. J. Janssen , Adv. Energy Mater. 2020, 10, 2000566.

[27] Z. Li , X. Li , M. Wang , M. Cai , X. Shi , Y. Mo , X. Chen , D. Ren , M. Yang , X. Liu , R. Jia , N. V. Medhekar , S. Dai , ACS Appl. Energy Mater. 2022, 5, 108.

[28] P. Y. Lin , C. F. Lin , Y. Y. Chiu , H. H. Chen , M. H. Li , R. Raja , C. S. Wu , C. H. Hou , S. Sung‐Yun Hsiao , J. J. Shyue , D. C. Lee , S. Z. Ho , Y. C. Chen , P. Chen , ACS Appl. Energy Mater. 2023, 6, 79.

[29] L. Wang , Z. Wang , H. Li , B. Chang , L. Pan , Z. Xie , L. Yin , ACS Appl. Mater. Interfaces 2022, 14, 18302. 35412305 10.1021/acsami.1c23949

[30] H. Dong , Z. Wu , J. Xi , X. Xu , L. Zuo , T. Lei , X. Zhao , L. Zhang , X. Hou , A. K. Jen , Adv. Funct. Mater. 2018, 28, 1704836.

[31] S. Mariotti , E. Köhnen , F. Scheler , K. Sveinbjörnsson , L. Zimmermann , M. Piot , F. Yang , B. Li , J. Warby , A. Musiienko , D. Menzel , F. Lang , S. Keßler , I. Levine , D. Mantione , A. Al‐ashouri , M. S. Härtel , K. Xu , A. Cruz , J. Kurpiers , P. Wagner , H. Köbler , J. Li , A. Magomedov , D. Mecerreyes , E. Unger , A. Abate , M. Stolterfoht , B. Stannowski , R. Schlatmann , et al., Science 2023, 381, 63. 37410849 10.1126/science.adf5872

[32] J. Liu , M. De Bastiani , E. Aydin , G. T. Harrison , Y. Gao , R. R. Pradhan , M. K. Eswaran , M. Mandal , W. Yan , A. Seitkhan , M. Babics , A. S. Subbiah , E. Ugur , F. Xu , L. Xu , M. Wang , A. ur Rehman , A. Razzaq , J. Kang , R. Azmi , A. A. Said , F. H. Isikgor , T. G. Allen , D. Andrienko , U. Schwingenschlögl , F. Laquai , S. De Wolf , Science 2022, 377, 302. 35737811 10.1126/science.abn8910

[33] W. H. M. Remmerswaal , B. T. van Gorkom , D. Zhang , M. M. Wienk , R. A. J. Janssen , Adv. Energy Mater. 2024, 14, 2303664. 10.1021/acsaem.4c01077 PMC11267499 39055068

[34] Y. Yang , H. Chen , C. Liu , J. Xu , C. Huang , C. D. Malliakas , H. Wan , A. S. R. Bati , Z. Wang , R. P. Reynolds , I. W. Gilley , S. Kitade , T. E. Wiggins , S. Zeiske , S. Suragtkhuu , M. Batmunkh , L. X. Chen , B. Chen , M. G. Kanatzidis , E. H. Sargent , Science 2024, 386, 898. 39571031 10.1126/science.adr2091

[35] X. Y. He , K. L. Wang , J. Chen , C. H. Chen , Y. Xia , L. Huang , R. J. Jin , N. Nizamani , Z. Su , X. Gao , Z. K. Wang , Small 2024, 21, 2410310. 10.1002/smll.202410310 39703151

[36] C. Liu , Y. Yang , H. Chen , J. Xu , A. Liu , A. S. R. Bati , H. Zhu , L. Grater , S. S. Hadke , C. Huang , V. K. Sangwan , T. Cai , D. Shin , L. X. Chen , M. C. Hersam , C. A. Mirkin , B. Chen , M. G. Kanatzidis , E. H. Sargent , Science 2023,. 382, 810. 37972154 10.1126/science.adk1633

[37] D. W. DeQuilettes , W. Zhang , V. M. Burlakov , D. J. Graham , T. Leijtens , A. Osherov , V. Bulovic , H. J. Snaith , D. S. Ginger , S. D. Stranks , Nat. Commun. 2016, 7, 11683. 27216703 10.1038/ncomms11683 PMC4890321

[38] I. Poli , F. Ambrosio , A. Treglia , F. J. Berger , M. Prato , M. D. Albaqami , F. De Angelis , A. Petrozza , F. De Angelis , A. Petrozza , Adv. Sci. 2022, 9, 2202795. 10.1002/advs.202202795 PMC9661860 36109174

[39] K. Datta , B. T. van Gorkom , Z. Chen , M. J. Dyson , T. P. A. van der Pol , S. C. J. Meskers , S. Tao , P. A. Bobbert , M. M. Wienk , R. A. J. Janssen , ACS Appl. Energy Mater. 2021, 4, 6650. 34337343 10.1021/acsaem.1c00707 PMC8317152

[40] S. G. Motti , M. Gandini , A. J. Barker , J. M. Ball , A. R. Srimath Kandada , A. Petrozza , ACS Energy Lett. 2016, 1, 726.

[41] S. Chen , X. Wen , S. Huang , F. Huang , Y. B. Cheng , M. Green , A. Ho‐Baillie , Sol. RRL 2017, 1, 1600001.

[42] S. G. Motti , D. Meggiolaro , A. J. Barker , E. Mosconi , C. A. R. Perini , J. M. Ball , M. Gandini , M. Kim , F. De Angelis , A. Petrozza , Nat. Photonics 2019, 13, 532.

[43] X. Zheng , X. Wang , W. Li , Z. Liu , W. Ming , H. Wang , H. Wang , D. Li , B. Liu , C. Yang , J. Phys. Chem. C 2021, 125, 19551.

[44] Z. Xu , X. Zhong , T. Hu , J. Hu , A. Kahn , B. P. Rand , J. Am. Chem. Soc. 2024, 146, 33368. 39585968 10.1021/jacs.4c08939

[45] T. P. A. van der Pol , K. Datta , M. M. Wienk , R. A. J. Janssen , Adv. Opt. Mater. 2022, 10, 2102557.

[46] F. Staub , I. Anusca , D. C. Lupascu , U. Rau , T. Kirchartz , J. Phys. Mater. 2020, 3, 025003.

[47] Y. Li , L. Li , L. Yang , Y. He , C. Zhang , T. Sun , M. Li , H. Zhao , X. Zhuang , Appl. Phys. Express 2023, 16, 062008.

[48] L. Gil‐Escrig , I. Susic , İ. Doğan , V. Zardetto , M. Najafi , D. Zhang , S. Veenstra , S. Sedani , B. Arikan , S. Yerci , H. J. Bolink , M. Sessolo , Adv. Funct. Mater. 2023, 33, 2214357.

[49] J. B. Patel , A. D. Wright , K. B. Lohmann , K. Peng , C. Q. Xia , J. M. Ball , N. K. Noel , T. W. Crothers , J. Wong‐leung , H. J. Snaith , L. M. Herz , M. B. Johnston , Adv. Energy Mater. 2020, 10, 1903653.

[50] R. K. Gunasekaran , J. Jung , S. W. Yang , D. Im , W. C. Choi , Y. Yun , S. Lee , ACS Energy Lett. 2024, 9, 102.

[51] G. Kapil , T. Bessho , T. Maekawa , A. K. Baranwal , Y. Zhang , M. A. Kamarudin , D. Hirotani , Q. Shen , H. Segawa , S. Hayase , Adv. Energy Mater. 2021, 11, 2101069.

[52] J. Wang , Y. Wu , J. Zhao , S. Lu , J. Lu , J. Sun , S. Wu , X. Zheng , X. Zheng , X. Tang , M. Ma , S. Yue , K. Liu , Z. Wang , S. Qu , Small Methods 2024, 8, 2400043. 10.1002/smtd.202400043 38462962

[53] J. Wang , L. Zeng , D. Zhang , A. Maxwell , H. Chen , K. Datta , A. Caiazzo , W. H. M. Remmerswaal , N. R. M. Schipper , Z. Chen , K. Ho , A. Dasgupta , G. Kusch , R. Ollearo , L. Bellini , S. Hu , Z. Wang , C. Li , S. Teale , L. Grater , B. Chen , M. M. Wienk , R. A. Oliver , H. J. Snaith , R. A. J. Janssen , E. H. Sargent , Nat. Energy 2024, 9, 70.

[54] K. Datta , J. Wang , D. Zhang , V. Zardetto , W. H. M. Remmerswaal , C. H. L. Weijtens , M. M. Wienk , R. A. J. Janssen , Adv. Mater. 2022, 34, 2110053. 10.1002/adma.202110053 34965005

[55] Q. Sun , X. Meng , J. Deng , B. Shen , D. Hu , B. Kang , S. R. P. Silva , J. Alloys Compd. 2023, 959, 170478.

