# Supplementary material for: Tailoring the Crystallization Behavior of Mixed Lead‐Tin Mixed‐Halide Perovskites for Optimal‐Bandgap Solar Cells
Source: Adv Sci (Weinh). 2025 Nov 19;13(7):e20948. doi: 10.1002/advs.202520948 (PMC12866725; doi:10.1002/advs.202520948)
Supplement: Supplementary file 1 — Supporting Information [file ADVS-13-e20948-s001.pdf]

Supporting information

Tailoring the Crystallization Behavior of Mixed Lead-Tin Mixed-Halide Perovskites for Optimal-Bandgap Solar Cells

Lana M. Kessels, Willemijn H. M. Remmerswaal, Nick R. M. Schipper, Laura Bellini, Henry Kwan, Martijn M. Wienk, and René A. J. Janssen

Table S1. Overview of the antisolvent procedures for various perovskite compositions related to dual-metal dual-halide compositions.

Composition	Δ*t* (s)	Antisolvent	E_g (eV)	PCE (%)	Ref.
$CsPb_{0.4}Sn_{0.6}I_{2.4}Br_{0.6}$	-	None	1.35	12.3	S1
$CsPb_{0.6}Sn_{0.4}I_2Br$	30	Ethyl acetate	1.54	14.2	S2
$MAPb_{0.4}Sn_{0.6}I_{2.6}Br_{0.4}$	5-6	Ether	1.25	12.1	S3
$Cs_{0.1}FA_{0.6}MA_{0.3}Sn_{0.5}Pb_{0.5}I_3$	20	Chlorobenzene	1.27	23.4	S4
$FAPb_{0.75}Sn_{0.25}I_3$	40	Chlorobenzene	1.38	17.3	S5
$FAPb_{0.5}Sn_{0.5}I_3$	18	Chlorobenzene	1.35	21.1	S6
$CsPb_{0.5}Sn_{0.5}I_2Br$	-	None	1.44	8.1	S7
$CsPb_{0.75}Sn_{0,25}IBr_2$	45	Chlorobenzene	1.78	11.5	S8
$FASnI_3$	10	Toluene	1.41	17.1	S9

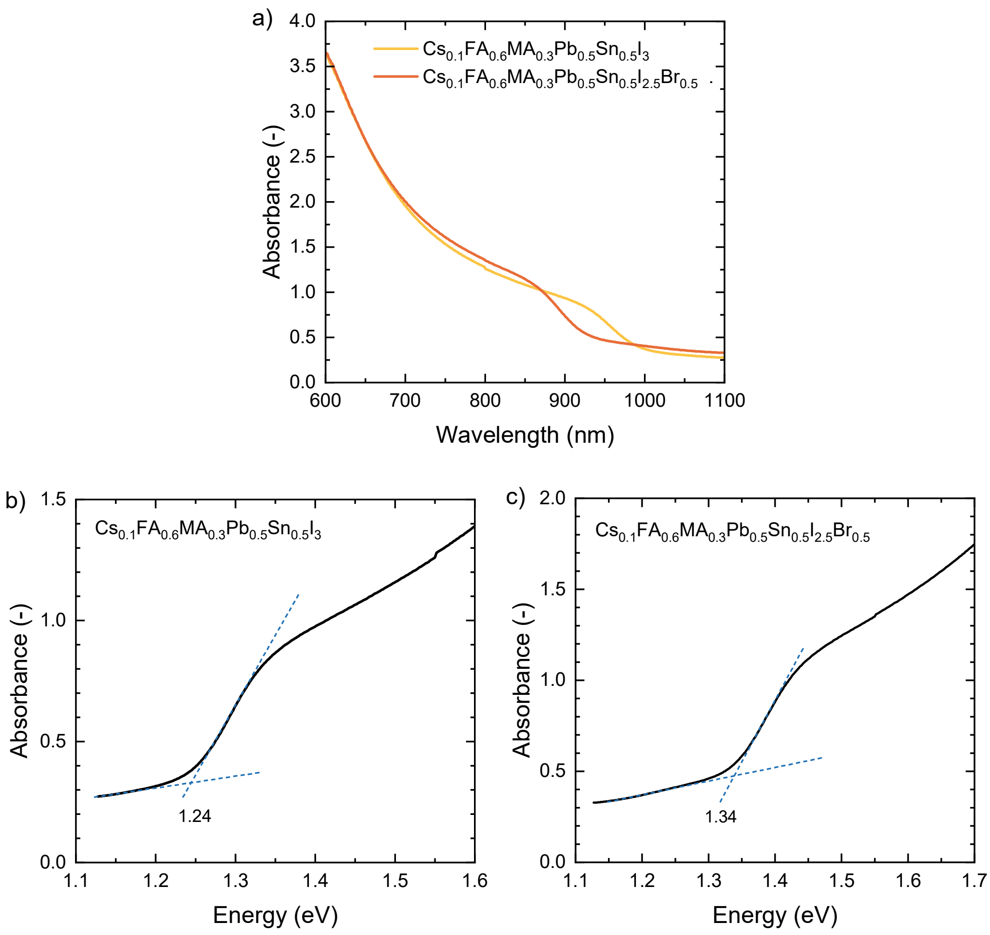

Figure S1. a) Absorbance spectra of $Cs_{0.1}FA_{0.6}MA_{0.3}Pb_{0.5}Sn_{0.5}I_3$ and $Cs_{0.1}FA_{0.6}MA_{0.3}Pb_{0.5}Sn_{0.5}I_{2.5}Br_{0.5}$ perovskites films. b) and c) Absorbance on energy scale to determine the bandgap of b) $Cs_{0.1}FA_{0.6}MA_{0.3}Pb_{0.5}Sn_{0.5}I_3$ and c) $Cs_{0.1}FA_{0.6}MA_{0.3}Pb_{0.5}Sn_{0.5}I_{2.5}Br_{0.5}$.

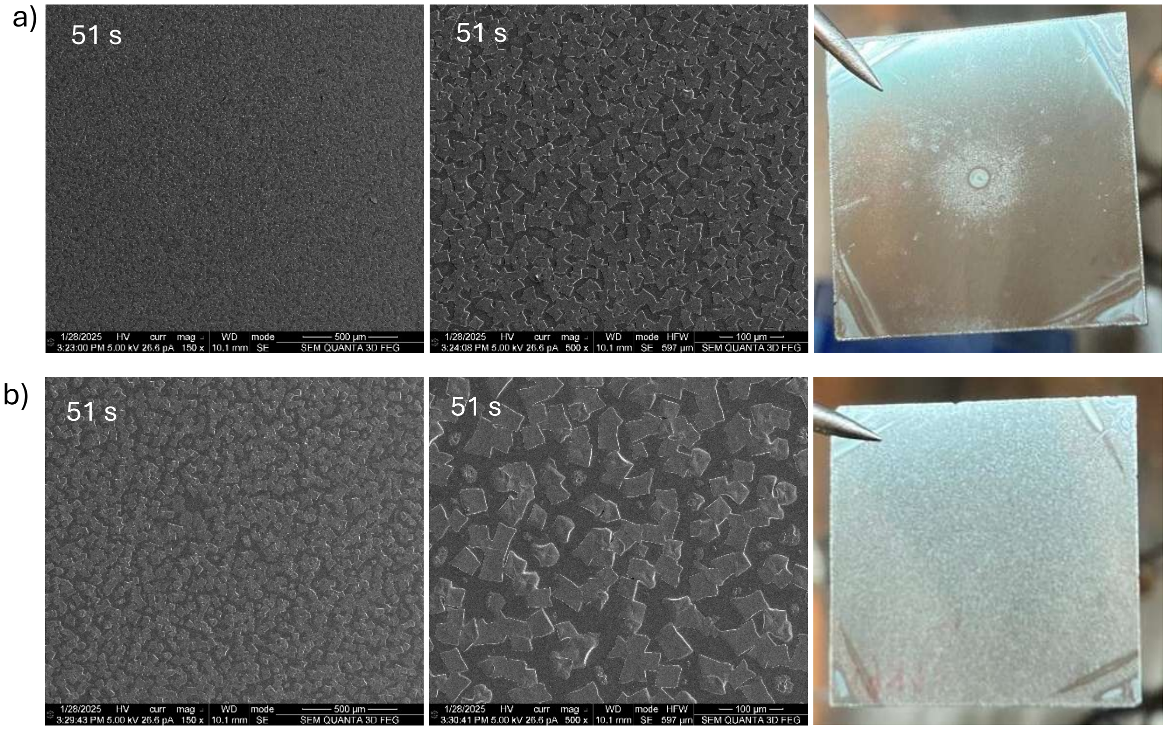

Figure S2. Scanning electron microscopy images and photos of $Cs_{0.1}FA_{0.6}MA_{0.3}Pb_{0.5}Sn_{0.5}I_{2.5}Br_{0.5}$ perovskite films made with the antisolvent dropping delay time of Δt = 51 s. a) and b) are from two different films. The scale bars are 500 (left) and 100 μm (middle).

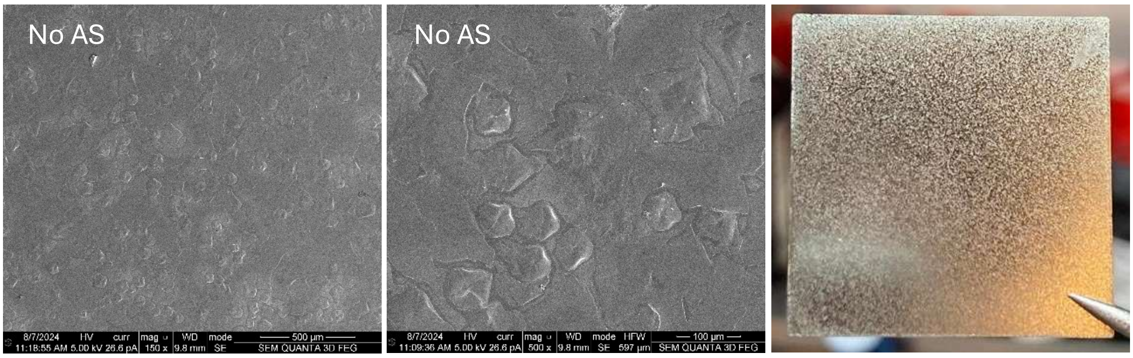

Figure S3. Scanning electron microscopy images and photo of a $Cs_{0.1}FA_{0.6}MA_{0.3}Pb_{0.5}Sn_{0.5}I_{2.5}Br_{0.5}$ perovskite film made without antisolvent. The scale bars are 500 (left) and 100 μm (middle).

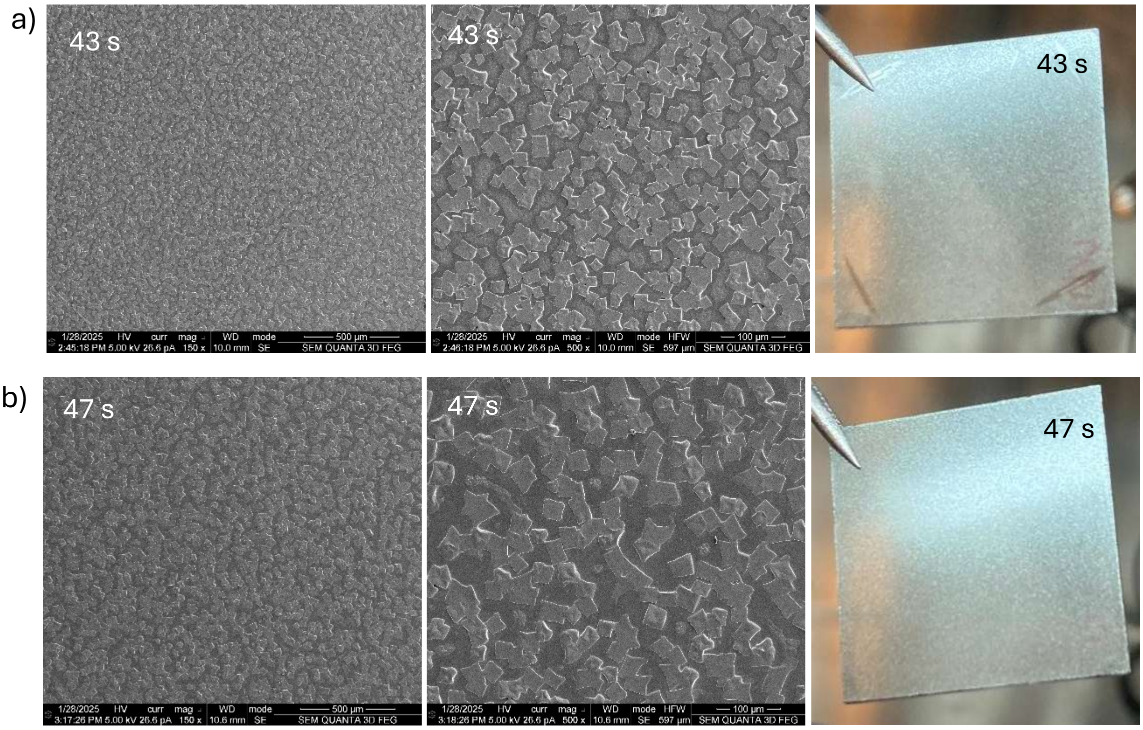

Figure S4. Scanning electron microscopy images and photos of $Cs_{0.1}FA_{0.6}MA_{0.3}Pb_{0.5}Sn_{0.5}I_{2.5}Br_{0.5}$ perovskite films made with antisolvent dropping delay times of a) Δt = 43 s and b) Δt = 47 s. The scale bars are 500 (left) and 100 μm (middle).

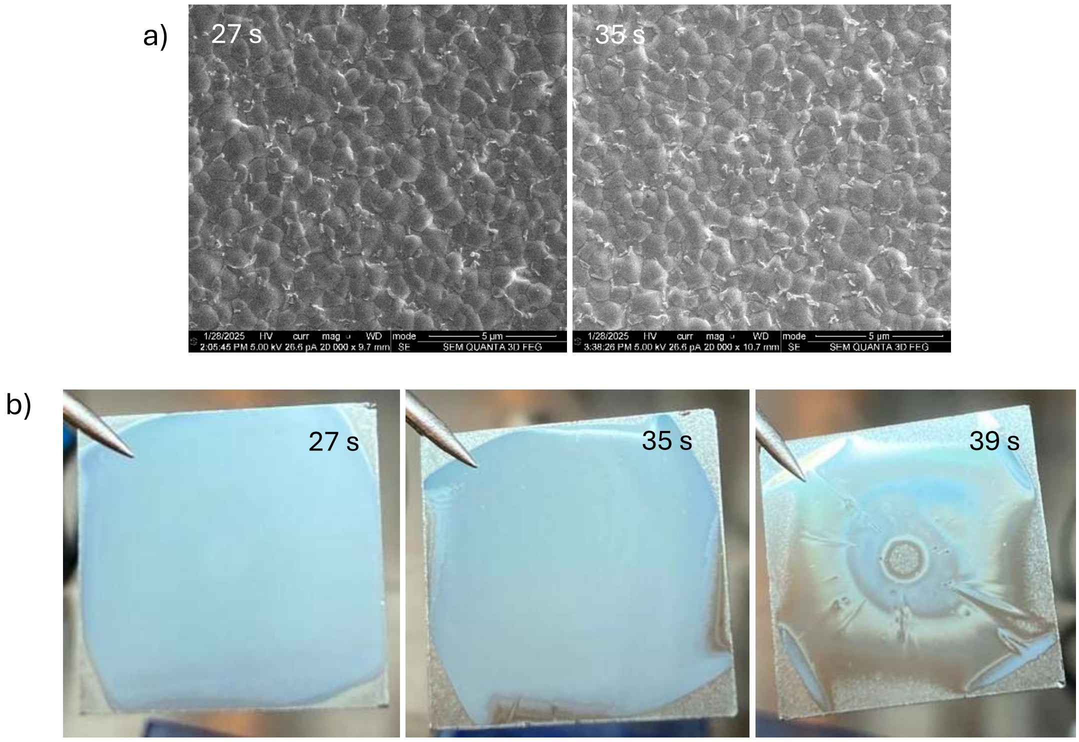

Figure S5. a) Scanning electron microscopy images and b) photos of $Cs_{0.1}FA_{0.6}MA_{0.3}Pb_{0.5}Sn_{0.5}I_{2.5}Br_{0.5}$ perovskite films made with the antisolvent delay times of Δt = 27, 35, and 39 s. The scale bars are 5 μm.

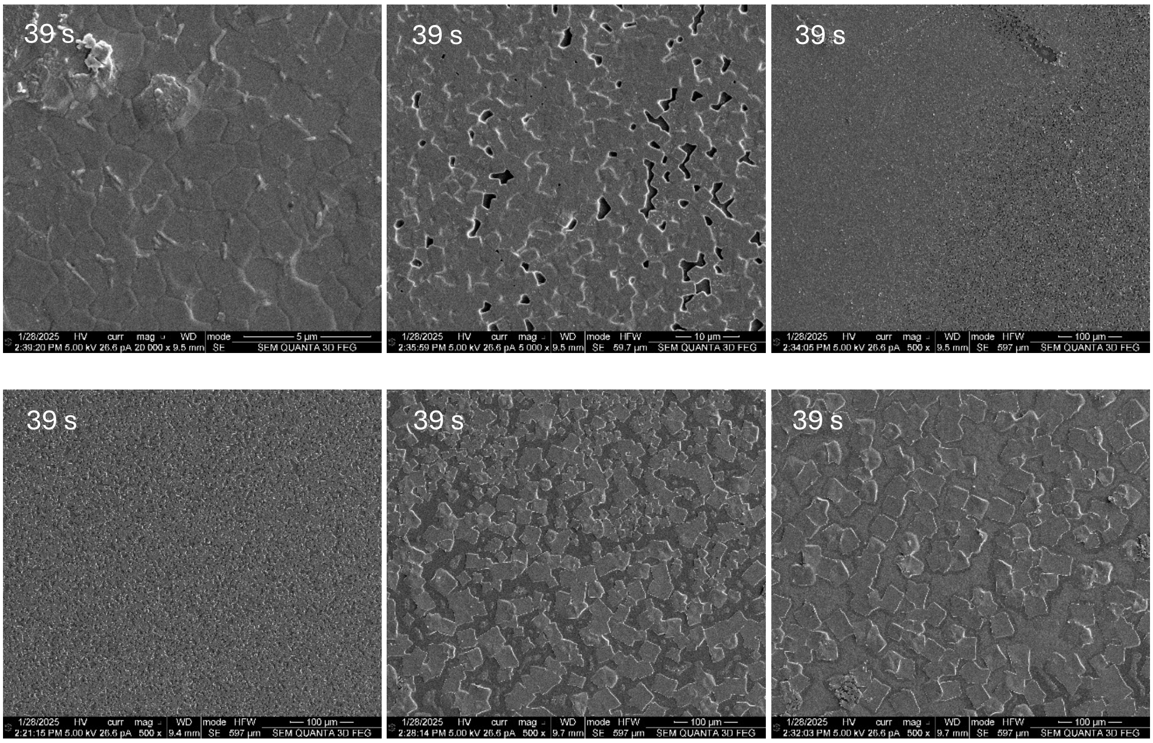

Figure S6. Scanning electron microscopy images of a $Cs_{0.1}FA_{0.6}MA_{0.3}Pb_{0.5}Sn_{0.5}I_{2.5}Br_{0.5}$ perovskite film made with the antisolvent delay times of Δt = 39 s, showing a variety of morphologies detected within the same film. The images have varying scale bars: 5 (top left), 10 (top middle), 100 µm (top right and bottom).

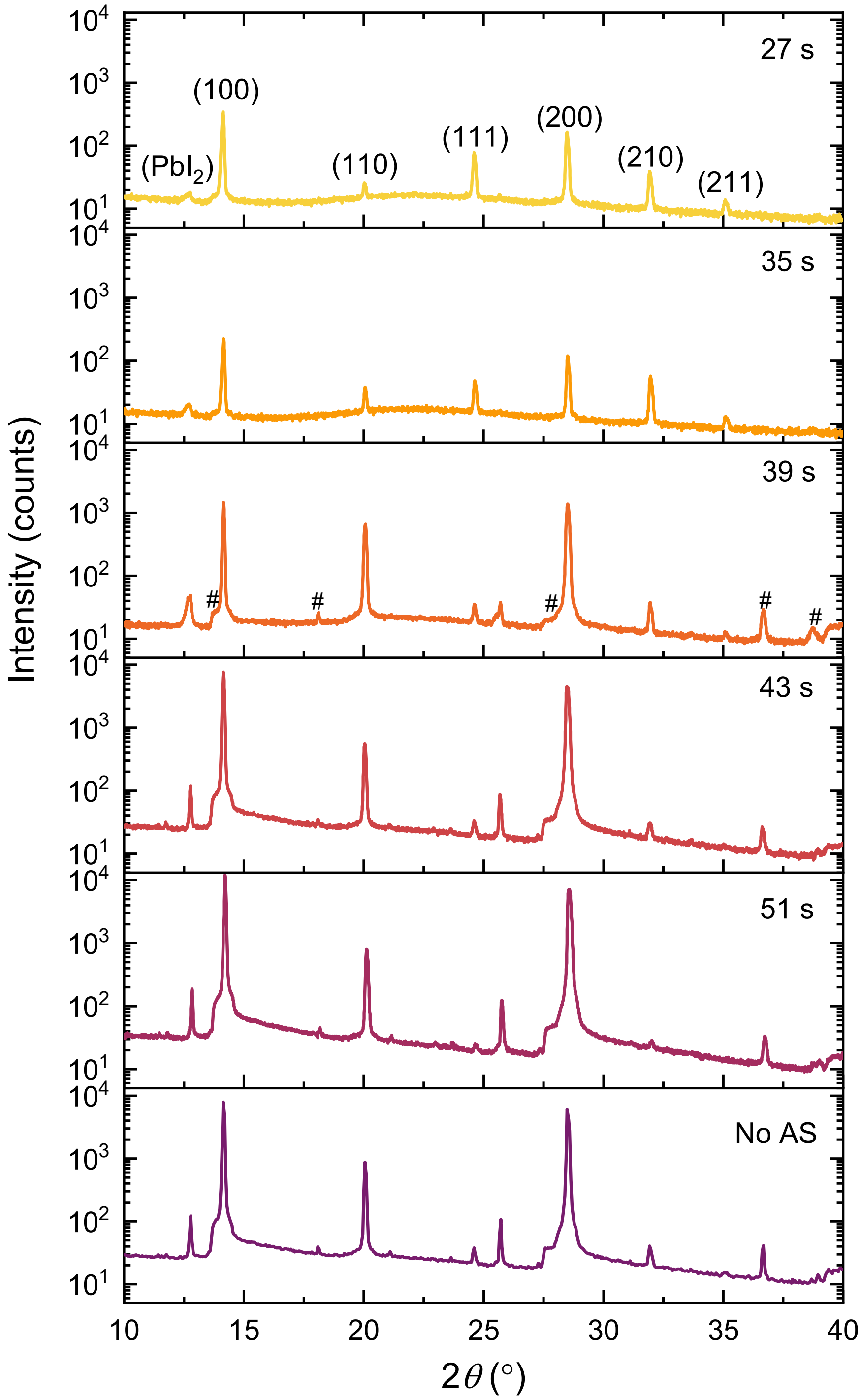

Figure S7. X-ray diffraction spectra of $Cs_{0.1}FA_{0.6}MA_{0.3}Pb_{0.5}Sn_{0.5}I_{2.5}Br_{0.5}$ perovskite films fabricated without (No AS) and with antisolvent dropping at Δt = 27, 35, 39, 43 and 51 s. Peaks indicated with a pound sign, present for $\Delta t \geq 39$ s could not be unambiguously assigned.

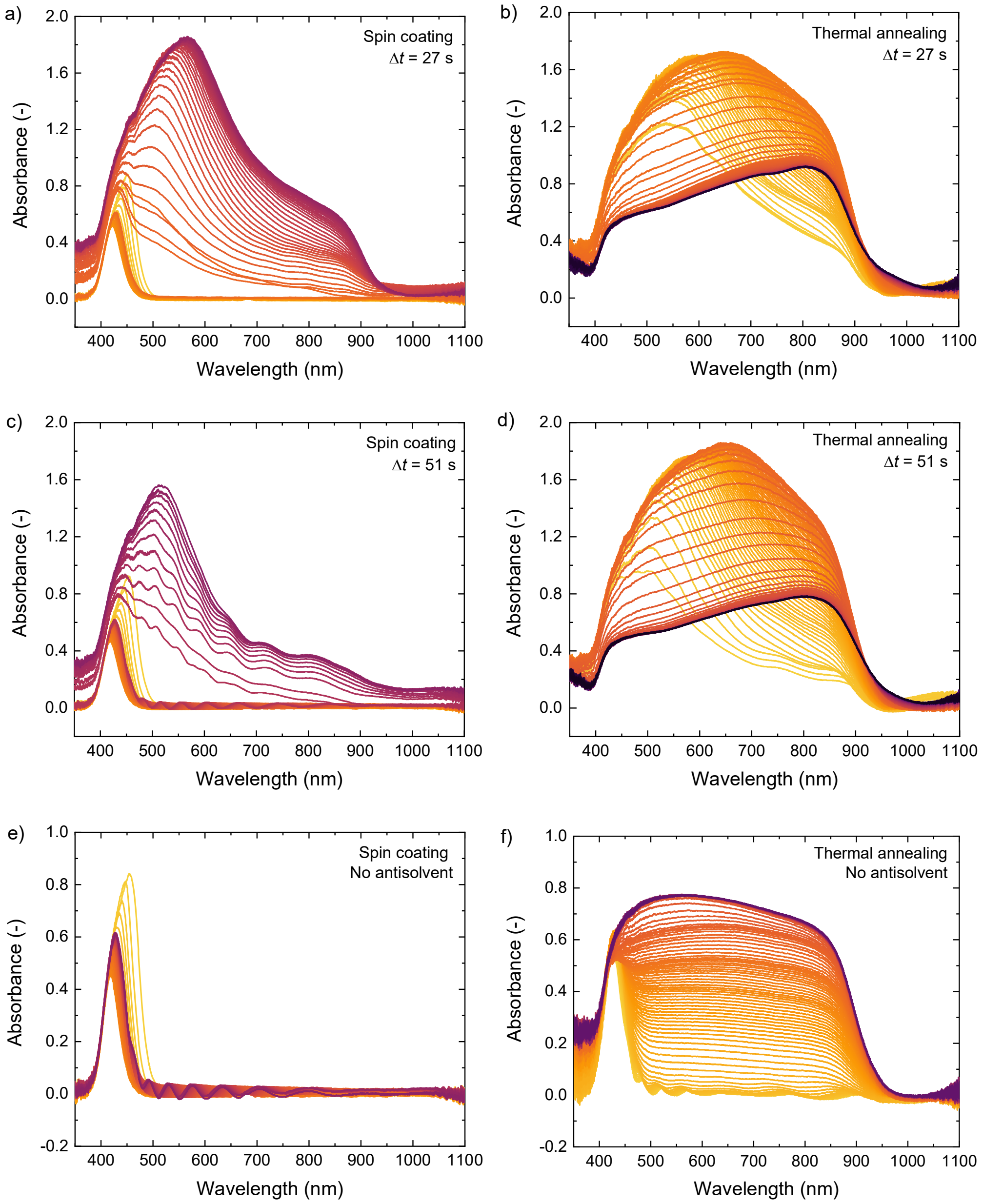

Figure S8. In-situ absorption measurements during spin-coating (a, c, e) and thermal annealing at 65 °C (b, d, f) of the perovskite layers made with Δt = 27 s (a, b) and 51 s (c, d), or without antisolvent (e, f). The plots show $A = -{}^{10}\log(I_s/I_b)$, where I_s and I_b represent the intensities of the light reflected from the sample (I_s) and from the blank (I_b) substrate, each after subtracting spurious background intensity. The progress in time is indicated from yellow to red and purple. Note the different vertical scale in panels e) and f).

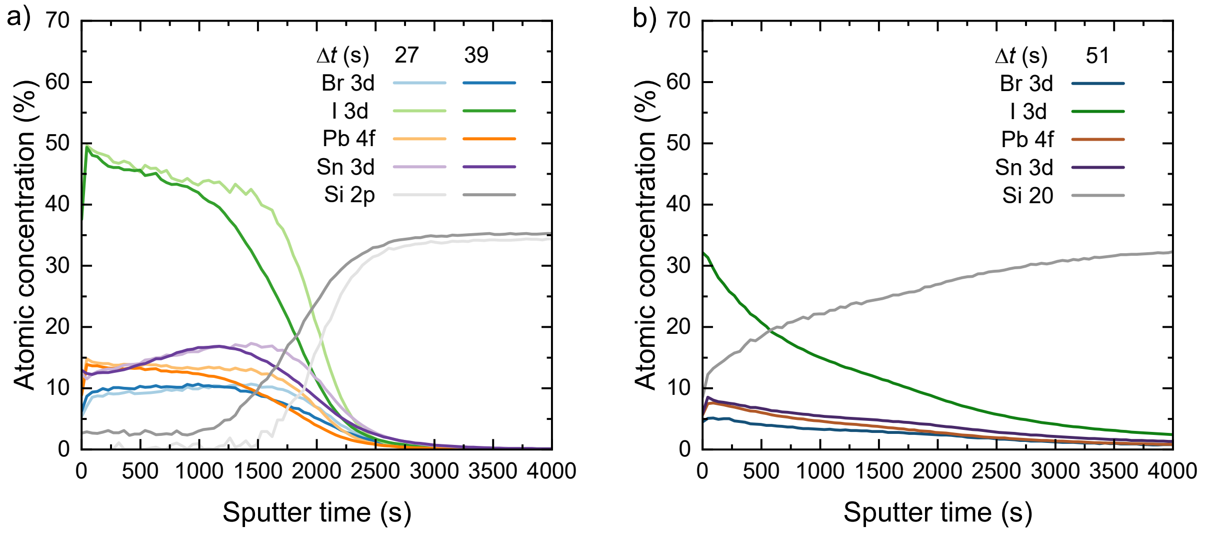

Figure S9. Atomic concentration depth profiles for Br 3d, I 3d, Pb 4f, Sn 3d, and Si 2p electrons from X-ray photoelectron spectroscopy (XPS) of $Cs_{0.1}FA_{0.6}MA_{0.3}Pb_{0.5}Sn_{0.5}I_{2.5}Br_{0.5}$ perovskite films made different delay times for antisolvent dropping. a) Δ*t* = 27 and 39 s. b) 51 s.

Table S2. Median and average grain size calculated from the grain size distribution of SEM images of perovskite films with 0, 1, 2, 4, 6, and 8 mol% NH_4SCN bulk additive.

Concentration NH_4SCN (mol%)	Median grain size (nm)	Mean grain size (nm)	Population size
0	666	701	145
1	964	953	87
2	1081	1113	120
4	1209	1212	100
6	1255	1255	140
8	1507	1516	107

Table S3. Profilometry measurements of perovskites films with 0, 1, 2, 4, 6, and 8 mol% NH_4SCN bulk additive.

Concentration NH_4SCN (mol%)	Thickness (nm)	Roughness (nm)
0	846 ± 26	22 ± 3
1	828 ± 4	14 ± 3
2	843 ± 2	21 ± 3
4	839 ± 10	22 ± 5
6	830 ± 18	23 ± 5
8	833 ± 29	25 ± 2

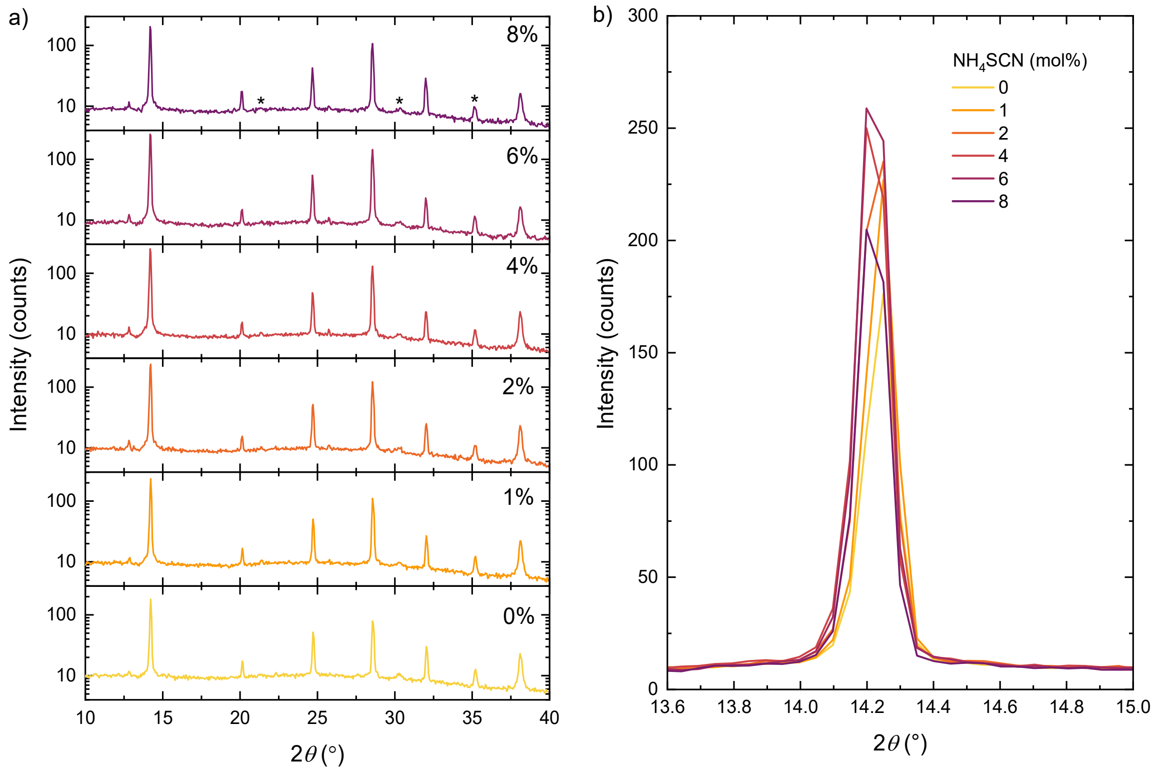

Figure S10. a) X-ray diffraction spectra of $Cs_{0.1}FA_{0.6}MA_{0.3}Pb_{0.5}Sn_{0.5}I_{2.5}Br_{0.5}$ perovskite films processed with 0, 1, 2, 4, 6, and 8 mol% NH_4SCN. The peaks indicated with an asterisk at 21.3°, 30.2°, and 38.2° are from the ITO glass substrate. b) Zoom-in of 14.2° region.

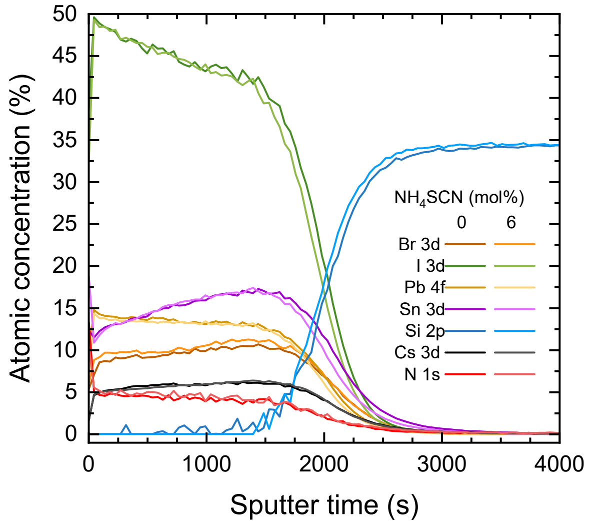

Figure S11. Atomic concentration depth profiles from X-ray photoelectron spectroscopy (XPS) of $Cs_{0.1}FA_{0.6}MA_{0.3}Pb_{0.5}Sn_{0.5}I_{2.5}Br_{0.5}$ perovskite films without (0) and with 6 mol% NH_4SCN as bulk additive for Br 3d, I 3d, Pb 4f, Sn 3d, Si 2p, Cs 3d, and N 1s electrons.

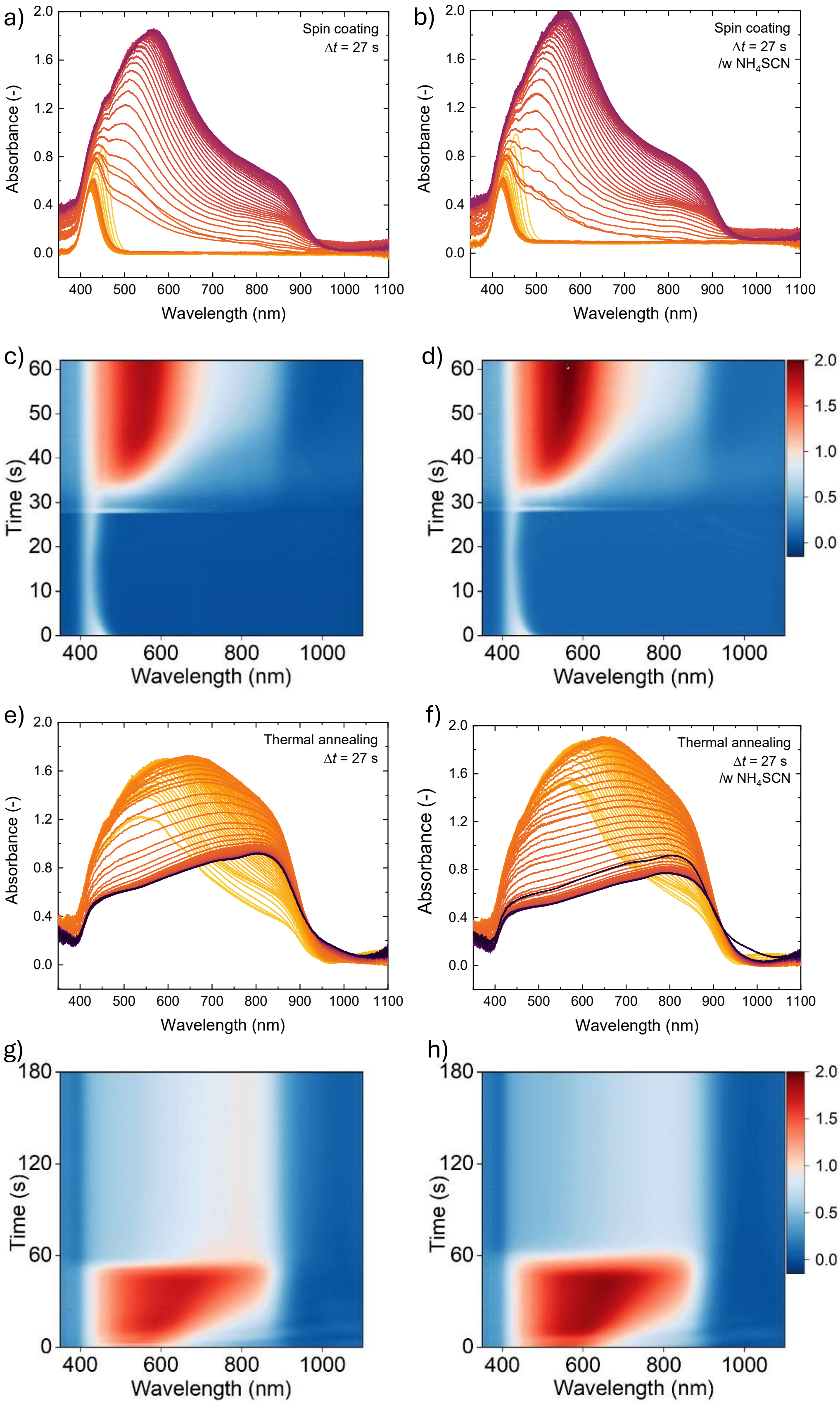

Figure S12. In-situ absorption measurement during spin-coating (panels a-d) and thermal annealing (panels e-h) for $Cs_{0.1}FA_{0.6}MA_{0.3}Pb_{0.5}Sn_{0.5}I_{2.5}Br_{0.5}$ perovskite layers processed without (a, c, e, g) and with NH_4SCN (b, d, f, h). In both experiments Δt = 27 s.

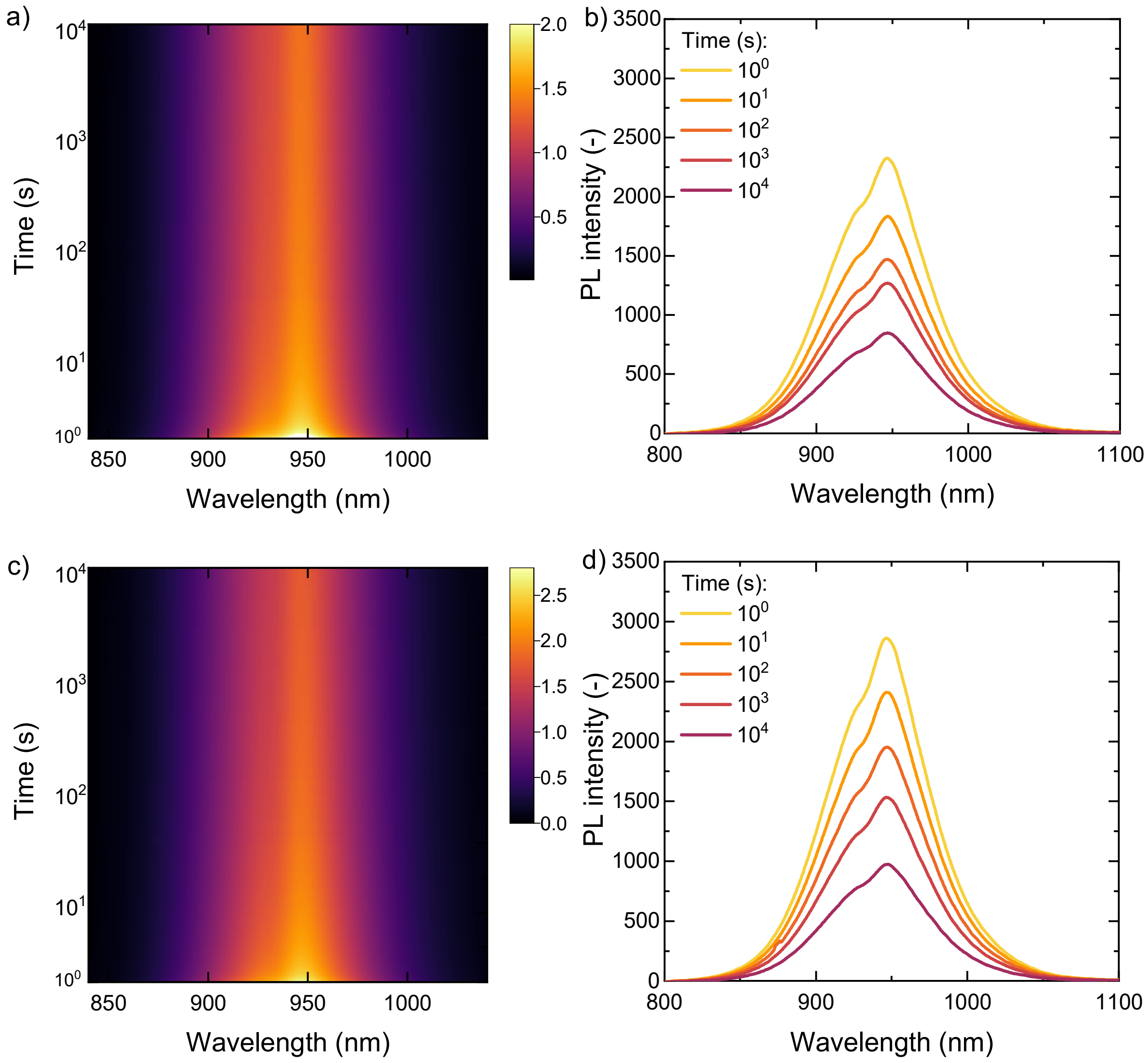

Figure S13. Photoluminescence over time during continuous 530 nm illumination at 1-sun equivalent intensity of $Cs_{0.1}FA_{0.6}MA_{0.3}Pb_{0.5}Sn_{0.5}I_{2.5}Br_{0.5}$ perovskite films processed with 6 mol% NH_4SCN. a, b) Without surface passivation. c, d) With $EDAI_2$ surface passivation.

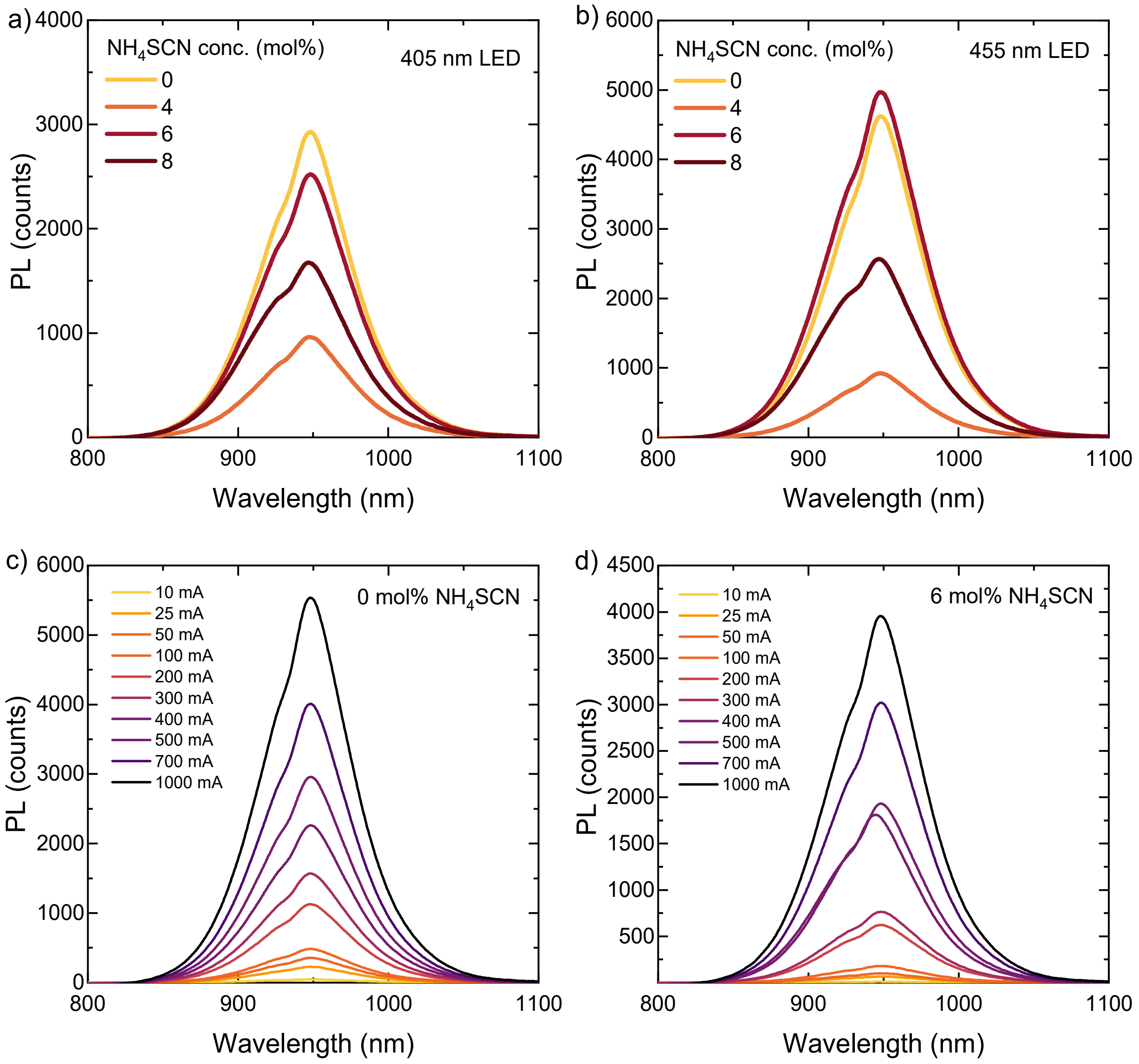

Figure S14. Absolute photoluminescence measured for $Cs_{0.1}FA_{0.6}MA_{0.3}Pb_{0.5}Sn_{0.5}I_{2.5}Br_{0.5}$ perovskite films on glass. a, b) For layers processed without and with 4, 6, and 8 mol% NH_4SCN with excitation at 405 nm a) or 455 nm b). c, d) Absolute photoluminescence measured at different light intensities with a 455 nm LED of layers processed without and with 6 mol% NH_4SCN.

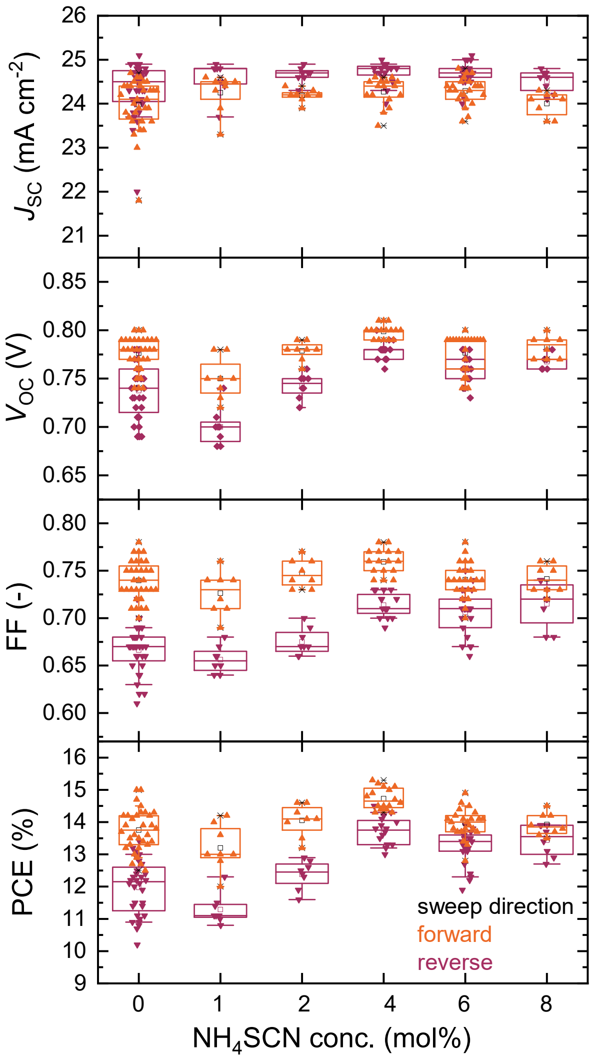

Figure S15. Statistics of the photovoltaic parameters determined from the current-density – voltage characteristics measured in forward (orange) and reverse (purple) sweep directions for ITO/PEDOT/$Cs_{0.1}FA_{0.6}MA_{0.3}Pb_{0.5}Sn_{0.5}I_{2.5}Br_{0.5}$/$C_{60}$/BCP/Ag solar cells processed with 0, 1, 2, 4, 6, and 8 mol% of NH_4SCN. The box represents the 25-75% region and the whiskers the 10-90% region.

Table S4. Device statistics of photovoltaic parameters of $Cs_{0.1}FA_{0.6}MA_{0.3}Pb_{0.5}Sn_{0.5}I_{2.5}Br_{0.5}$ perovskite solar cells processed with 0, 1, 2, 4, 6, or 8 mol% NH_4SCN.

NH_4SCN	Scan	J_{SC} (mA cm^{-2})	V_{OC} (V)	FF (-)	PCE (%)
0 mol%	Backward	24.3 ± 0.6	0.74 ± 0.03	0.67 ± 0.02	12.0 ± 0.8
	Forward	24.0 ± 0.6	0.78 ± 0.02	0.74 ± 0.02	13.8 ± 0.6
1 mol%	Backward	24.6 ± 0.4	0.70 ± 0.02	0.66 ± 0.01	11.3 ± 0.5
	Forward	24.3 ± 0.4	0.75 ± 0.02	0.73 ± 0.02	13.2 ± 0.7
2 mol%	Backward	24.7 ± 0.2	0.74 ± 0.01	0.68 ± 0.01	12.4 ± 0.4
	Forward	24.2 ± 0.1	0.78 ± 0.01	0.75 ± 0.01	14.1 ± 0.5
4 mol%	Backward	24.7 ± 0.3	0.78 ± 0.01	0.71 ± 0.01	13.7 ± 0.4
	Forward	24.3 ± 0.3	0.80 ± 0.01	0.76 ± 0.01	14.7 ± 0.3
6 mol%	Backward	24.7 ± 0.3	0.76 ± 0.01	0.71 ± 0.02	13.2 ± 0.5
	Forward	24.3 ± 0.3	0.78 ± 0.02	0.74 ± 0.02	14.0 ± 0.4
8 mol%	Backward	24.5 ± 0.3	0.77 ± 0.01	0.72 ± 0.02	13.4 ± 0.5
	Forward	24.0 ± 0.3	0.78 ± 0.01	0.74 ± 0.02	13.9 ± 0.3

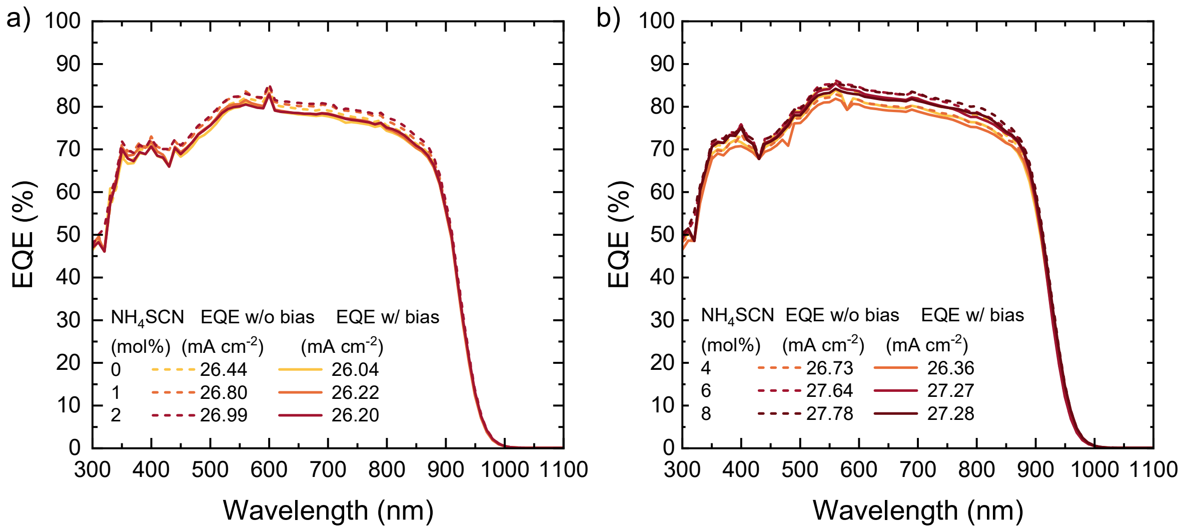

Figure S16. External quantum efficiencies of $Cs_{0.1}FA_{0.6}MA_{0.3}Pb_{0.5}Sn_{0.5}I_{2.5}Br_{0.5}$ solar cells measured without and with 530 nm 1-sun equivalent light bias processed with a) 0, 1, and 4 mol% NH_4SCN, and b) 4, 6, and 8 mol% NH_4SCN. The corresponding EQE-integrated short-circuit current-densities are given in the legends. Note that the J_{SC} determined from the *J-V* measurement (Table S4) underestimates the EQE-integrated $J_{SC,EQE}$ about 10% as a result of the light-source used (see Experimental Section for details).

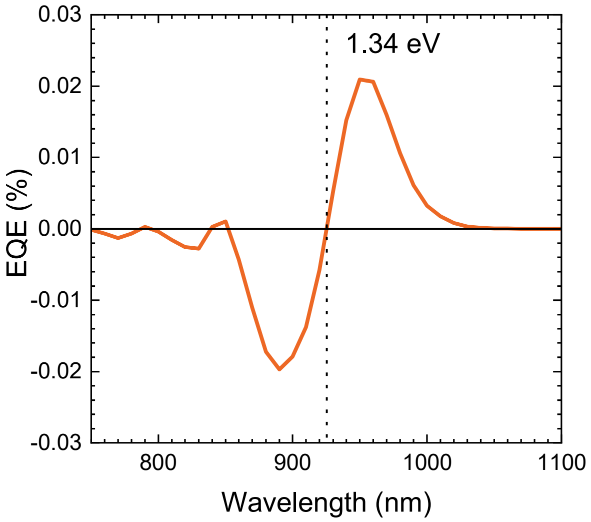

Figure S17. Second derivative of the EQE spectrum of a $Cs_{0.1}FA_{0.6}MA_{0.3}Pb_{0.5}Sn_{0.5}I_{2.5}Br_{0.5}$ perovskite solar cell processed with 6 mol% NH_4SCN, $EDAI_2$ interface passivation, a thin PEDOT:PSS HTL, and an antireflective coating.

Table S5. Performance parameters of perovskite single-junction solar cells with the highest efficiency as a function of device absorber bandgap (E_g) in the 1.3 to 1.4 eV range.[S10] [a]

E_g [eV]	PCE [%]	V_{oc} [mV]	J_{sc} [mA cm^{-2}]	FF [%]	Absorber	Ref.
1.30	18.8	820	29.6	77.3	$FA_{0.6}MA_{0.4}Pb_{0.4}Sn_{0.6}I_3$	S11
1.30	17.1	840	27.9	73.0	$Cs_{0.05}FA_{0.8}MA_{0.15}Pb_{0.5}Sn_{0.5}I_3$	S12
1.31	5.0	420	23.8	50.3	$CsSnI_3$	S13
1.31	7.1	486	22.9	64.0	$MASnI_3$	S14
1.31	14.1	740	26.7	71.4	$Cs_{0.25}FA_{0.75}Pb_{0.5}Sn_{0.5}I_3$	S15
1.32	11.6	720	23.4	68.9	$MAPb_{0.4}Sn_{0.6}Br_{0.2}I_{2.8}$	S16
1.33	7.5	450	24.9	67.0	$CsSnI_3$:MBAA	S17
1.33	22.5	899	30.9	81.2	$FA_{0.8}MA_{0.2}Pb_{0.8}Sn_{0.2}I_3$	S18 [b]
1.34	10.0	767	20.5	63.6	$MAPb_{0.4}Sn_{0.6}I_3$	S19
1.34	12.1	780	20.7	75.1	$MAPb_{0.4}Sn_{0.6}Br_{0.4}I_{2.6}$	S16
**1.34** [c]	**19.0**	**866**	**28.4**	**77.3**	**$Cs_{0.1}FA_{0.6}MA_{0.3}Pb_{0.5}Sn_{0.5}I_{2.5}Br_{0.5}$**	
1.35	21.1	846	31.4	79.5	$FAPb_{0.5}Sn_{0.5}I_3$	S6
1.35	16.3	780	26.5	79.0	$FAPb_{0.7}Sn_{0.3}I_3$	S20
1.35	12.3	860	19.0	75.1	$CsPb_{0.4}Sn_{0.6}I_{2.4}Br_{0.6}$	S1 [b]
1.36	8.2	630	19.7	66.1	$CsSnI_3$	S21
1.37	14.7	737	27.1	73.6	$FA_{0.3}MA_{0.7}Pb_{0.7}Sn_{0.3}I_3$	S22
1.38	17.3	810	28.2	75.4	$FAPb_{0.75}Sn_{0.25}I_3$	S5
1.38	15.2	800	26.2	72.5	$MAPb_{0.75}Sn_{0.25}I_3$	S23
1.39	20.6	1020	26.6	76.0	$FA_{0.7}MA_{0.3}Pb_{0.7}Sn_{0.3}I_3$	S24
1.40	15.4	856	24.8	72.4	$FA_{0.85}PEA_{0.15}SnI_3$	S25

[a] The table is based on Ref. [10] where the device absorber bandgap energy is determined from the EQE spectrum. [b] Entry is not part of Ref [10] its E_g value is based on a Tauc plot. [c] This work.

Supplementary references

[S1] S. Lee, J. Moon, J. Ryu, B. Parida, S. Yoon, D.-G. Lee, J. S. Cho, S. Hayase, D.-W. Kang, *Nano Energy* **2020**, *77*, 105309.

[S2] Q. Wen, C Duan, F. Zou, D. Luo, J. Li, Z. Liu, J. Wang, K. Yan, *Chem. Eng. J.* **2023**, *452*, 139697.

[S3] S. Lee , D.-W. Kang, *ACS Appl. Mater. Interfaces* **2017**, *9*, 22432.

[S4] J. Zhao, Z. Su, J. Pascual, H. Wu, H. Wang, M. H. Aldamasy, Z. Zhou, C. Wang, G. Li, Z. Li, X. Gao, C.-S. Hsu, M. Li, *Adv. Mater.* **2024**, *36*, 2406246.

[S5] D. Chi, S. Huang, M. Zhang, S. Mu, Y. Zhao, Y. Chen, J. You, *Adv. Funct. Mater.* **2018**, *28*, 1804603.

[S6] Y. Zhou, T. Guo, J. Jin, Z. Zhu, Y. Li, S. Wang, S. Zhou, Q. Lin, J. Li, W. Ke, G. Fang, X. Zhang, Q. Tai, *Energy Environ. Sci.* **2024**, *17,* 2845.

[S7] H. Ban, Q. Sun, T. Zhang, H. Li, Y. Shen, M. Wang, *Sol. RRL* **2020**, *4*, 1900457.

[S8] N. Li, Z. Zhu, J. Li, A. K.-Y. Jen, L. Wang. *Adv. Energy Mater*. **2018**, *8*, 1800525.

[S9] D. He, Peng Chen, J. A. Steele, Z. Wang, H. Xu, M. Zhang, S. Ding, C. Zhang, T. Lin, F. Kremer, H. Xu, M. Hao, L. Wang, *Nat. Nanotechnology,* **2025**, *20*, 779.

[S10] O. Almora, G. C. Bazan, C. I. Cabrera, L. A. Castriotta, S. Erten-Ela, K. Forberich, K. Fukuda, F. Guo, J. Hauch, A. W. Y. Ho-Baillie, T. J. Jacobsson, R. A. J. Janssen, T. Kirchartz, R. R. Lunt, X. Mathew, D. B. Mitzi, M. K. Nazeeruddin, J,. Nelson, A. F. Nogueira, U. W. Paetzold, B. P. Rand, U. Rau, T. Someya, C. Sprau, E. Unger, L. Vaillant-Roca, C. J. Brabec, *Adv. Energy Mater.* **2025**, *15*, 2404386.

[S11] H. Chen, Z. Peng, K. Xu, Q. Wei, D. Yu, C. Han, H. Li, Z. Ning, *Sci. China Mater.* **2021**, *64*, 537.

[S12] N. Ghimire, R. S. Bobba, A. Gurung, K. M. Reza, M. A. R. Laskar, B. S. Lamsal, K. Emshadi, R. Pathak, M. A. Afroz, A. H. Chowdhury, K. Chen, B. Bahrami, S. I. Rahman, J. Pokharel, A. Baniya, M. T. Rahman, Y. Zhou, Q. Qiao, *ACS Appl. Energy Mater.* **2021**, *4*, 1731.

[S13] Y. Wang, J. Tu, T. Li, C. Tao, X. Deng, Z. Li, *J. Mater. Chem. A* **2019**, *7*, 7683.

[S14] F. Li, C. Zhang, J.-H. Huang, H. Fan, H. Wang, P. Wang, C. Zhan, C.-M. Liu, X. Li, L.-M. Yang, Y. Song, K.-J. Jiang, *Angew. Chem., Int. Ed.* **2019**, *58*, 6688.

[S15] G. E. Eperon, T. Leijtens, K. A. Bush, R. Prasanna, T. Green, J. T.-W. Wang, D. P. McMeekin, G. Volonakis, R. L. Milot, R. May, A. Palmstrom, D. J. Slotcavage, R. A. Belisle, J. B. Patel, E. S. Parrott, R. J. Sutton, W. Ma, F. Moghadam, B. Conings, A. Babayigit, H.-G. Boyen, S. Bent, F. Giustino, L. M. Herz, M. B. Johnston, M. D. McGehee, H. J. Snaith, *Science* **2016**, *354*, 861.

[S16] S. Lee, D.-W. Kang, *ACS Appl. Mater. Interfaces* **2017**, *9*, 22432.

[S17] T. Ye, K. Wang, Y. Hou, D. Yang, N. Smith, B. Magill, J. Yoon, R. R. H. H. Mudiyanselage, G. A. Khodaparast, K. Wang, S. Priya, *J. Am. Chem. Soc.* **2021**, *143*, 4319.

[S18] Z. Liang, H. Xu, Y. Zhang, G. Liu, S. Chu, Y. Tao, X. Xu, S. Xu, L. Zhang, X. Chen, B. Xu, Z. Xiao, X. Pan, J. Ye, *Adv. Mater.* **2022**, *34*, 2110241

[S19] B. Zhao, M. Abdi-Jalebi, M. Tabachnyk, H. Glass, V. S. Kamboj, W. Nie, A. J. Pearson, Y. Puttisong, K. C. Gödel, H. E. Beere, D. A. Ritchie, A. D. Mohite, S. E. Dutton, R. H. Friend, A. Sadhanala, *Adv. Mater.* **2017**, *29*, 1604744.

[S20] X. Lian, J. Chen, Y. Zhang, M. Qin, J. Li, S. Tian, W. Yang, X. Lu, G. Wu, H. Chen, *Adv. Funct. Mater.* **2019**, *29*, 1807024.

[S21] B. Li, H. Di, B. Chang, R. Yin, L. Fu, Y.-N. Zhang, L. Yin, *Adv. Funct. Mater.* **2021**, *31*, 2007447.

[S22] M. Zhang, D. Chi, J. Wang, F. Wu, S. Huang, *Sol. Energy* **2020**, *201*, 589.

[S23] H. L. Zhu, J. Xiao, J. Mao, H. Zhang, Y. Zhao, W. C. H. Choy, *Adv. Funct. Mater.* **2017**, *27*, 1605469.

[S24] X. Zhou, L. Zhang, X. Wang, C. Liu, S. Chen, M. Zhang, X. Li, W. Yi, B. Xu, *Adv. Mater.* **2020**, *32*, 1908107.

[S25] J. Chen, J. Luo, E. Hou, P. Song, Y. Li, C. Sun, W. Feng, S. Cheng, H. Zhang, L. Xie, C. Tian, Z. Wei, *Nat. Photon.* **2024**, *18*, 464.
